# Sox genes in the coral *Acropora millepora*: divergent expression patterns reflect differences in developmental mechanisms within the Anthozoa

**DOI:** 10.1186/1471-2148-8-311

**Published:** 2008-11-12

**Authors:** Chuya Shinzato, Akira Iguchi, David C Hayward, Ulrich Technau, Eldon E Ball, David J Miller

**Affiliations:** 1 ARC Centre of Excellence for Coral Reef Studies and Comparative Genomics Centre, James Cook University, Townsville, Queensland 4811, Australia; 2 ARC Centre for the Molecular Genetics of Development, Research School of Biological Sciences, Australian National University, PO Box 475, Canberra, ACT 2601, Australia; 3 Faculty of Life Sciences, University of Vienna, Althanstrasse 14, 1090 Wien, Austria; 4 Okinawa Institute of Science and Technology, Urama, Okinawa 904-2234, Japan; 5 Graduate School of Engineering and Science, University of the Ryukyus, Nishihara, Okinawa 903-0213, Japan

## Abstract

**Background:**

Sox genes encode transcription factors that function in a wide range of developmental processes across the animal kingdom. To better understand both the evolution of the Sox family and the roles of these genes in cnidarians, we are studying the *Sox *gene complement of the coral, *Acropora millepora *(Class Anthozoa).

**Results:**

Based on overall domain structures and HMG box sequences, the *Acropora Sox *genes considered here clearly fall into four of the five major Sox classes. *AmSoxC *is expressed in the ectoderm during development, in cells whose morphology is consistent with their assignment as sensory neurons. The expression pattern of the *Nematostella *ortholog of this gene is broadly similar to that of *AmSoxC*, but there are subtle differences – for example, expression begins significantly earlier in *Acropora *than in *Nematostella*. During gastrulation, *AmSoxBb *and *AmSoxB1 *transcripts are detected only in the presumptive ectoderm while *AmSoxE1 *transcription is restricted to the presumptive endoderm, suggesting that these *Sox *genes might play roles in germ layer specification. A third type B *Sox *gene, *AmSoxBa*, and a *Sox *F gene *AmSoxF *also have complex and specific expression patterns during early development. Each of these genes has a clear *Nematostella *ortholog, but in several cases the expression pattern observed in *Acropora *differs significantly from that reported in *Nematostella*.

**Conclusion:**

These differences in expression patterns between *Acropora *and *Nematostella *largely reflect fundamental differences in developmental processes, underscoring the diversity of mechanisms within the anthozoan Sub-Class Hexacorallia (Zoantharia).

## Background

*Sox *genes encode a family of transcription factors that are defined by the presence of an HMG box resembling that of the human testis determinant, *SRY*. Sox transcription factors are restricted to the animal kingdom, but are highly diversified and widely distributed within it. Mouse and man have 20 *Sox *genes [1] whereas the model invertebrates *Caenorhabditis *and *Drosophila *have much less extensive *Sox *repertoires (5 and 8 genes respectively; [2-4]). Despite the diversity and heterogeneity of *Sox *genes, there are some cases of apparent orthology between *Drosophila *and mammals. For example, the similarity in HMG box sequences of group B1 *Sox *genes between *Drosophila *and vertebrates, and the linkage of *Dichaete *(a putative group B2 *Sox *gene) to the B1 type gene *SoxNeuro *in *Drosophila *suggests the possibility that this organization dates back to the common bilaterian ancestor [5]. *Sox *genes have also been identified in the genomes of the placozoan *Trichoplax*, sponges, ctenophores and cnidarians [6-10].

In the most extensive evolutionary analysis to date [2], ten Sox groups (A-J) are recognized based on HMG domain sequences as well as structural characteristics such as intron-exon organization. Some of these groups have a wide phylogenetic distribution (SoxB-F), whereas others are restricted to specific lineages (SoxA and G-I to vertebrates; SoxJ to *Caenorhabditis*). The HMG domain sequences (79 amino acid residues) are generally very similar within a Sox group, but across deep phylogenetic divides (such as between *Drosophila *and vertebrates), the remainder of the protein sequence varies much more. However, between different vertebrate species, there is typically more extensive overall similarity between members of the same Sox group; not only are the HMG domains similar, but other functional domains are conserved and sometimes diagnostic for specific Sox groups/subgroups. Where members of the same Sox group have common features beyond the HMG domain, this conservation tends to correlate with similarity of function [2].

*Sox *genes play a variety of developmental roles, many of which are taxon-specific. Such functions include the role in which *Sox *genes were originally identified – testis determination by *SRY *– a function conserved only amongst eutherian mammals [11,12]. In a few specific cases, however, functions of particular Sox types/subtypes appear to be conserved between vertebrates and *Drosophila*. For example, group B *Sox *genes are expressed in developing neural tissues in a wide range of animals [4], and functional analyses imply that members of this group play central roles in early neuroectoderm differentiation in *Drosophila *and vertebrates [13-15]. In both *Xenopus *and *Drosophila*, *SoxB1 *type genes (*Sox2 *and *SoxNeuro *respectively) are essential for secondary steps of neural differentiation; and these are down- and up-regulated by the TGFb superfamily growth factor *dpp*/*BMP4 *and its inhibitor *sog*/*Chordin *[14,16]. Group B *Sox *genes are also expressed in neural tissues in other invertebrates, including amphioxus, hemichordates, ascidians and molluscs [17-21]. Taken together, these lines of evidence suggest that the involvement of group B *Sox *genes in early nervous system development may be an ancestral characteristic in the Bilateria. In addition to roles in the nervous system, group B *Sox *genes function in germ layer differentiation during early embryogenesis in both vertebrates and invertebrates. For example, *Sox3 *(group B) genes regulate gastrulation and germ layer formation in *Xenopus *and zebrafish [22], and in the sea urchin, maternal *SoxB1 *and *B2 *are required for gastrulation and vegetal development [23].

As anthozoan cnidarians are traditionally considered to be amongst the simplest animals at the tissue level of organization and have the simplest known nervous systems, they are an important comparator for establishing ancestral roles of genes and understanding the evolution of metazoan gene families. Earlier surveys [6,9] have demonstrated unexpected *Sox *gene diversity in non-Bilaterians, some diversification having occurred prior to the sponge divergence, and more extensive expansion predating the cnidarian divergence. Four *Sox *genes have been reported from the sponge *Amphimedon *(formerly *Reniera*; [7,8]), and a total of fourteen sequences from the sea anemone *Nematostella vectensis *[9]. Demosponge sequences fall into the B, C and E/F Sox type clades in the analyses of Jager et al. [6]; the analyses presented by Magie et al. [9] place the *Nematostella *genes into a total of six of the ten recognized Sox types. It is becoming clear that, like other animals (e.g. [24]), *Nematostella *has undergone secondary gene loss; for example, in the Forkhead transcription factor family [25]. Data for other cnidarians are therefore required to better understand the evolution of metazoan gene families such as the *Sox *genes. To this end, we have characterized *Sox *genes from a second anthozoan cnidarian, the staghorn coral *Acropora millepora*. These include members of the SoxB, C, E and F types; evaluation of all of the available data suggests that the SoxA, D, G, H, I and J types are restricted to the Bilateria. Some of the *Acropora Sox *genes are expressed in patterns that are consistent with roles in early germ layer development, and the *SoxC *gene is expressed in presumed sensory neurons. *Acropora *and *Nematostella *are often considered "close" relatives and, as they belong to the same Sub-Class (Hexacorallia) within the cnidarian Class Anthozoa, similar gene expression patterns might be expected. However, this is not always the case – the expression patterns of several of the *Acropora Sox *genes differ substantially from those of their likely orthologs in *Nematostella*. This apparent paradox is, however, largely a consequence of morphological differences and divergent developmental mechanisms between the coral and sea anemone. Given their extensive developmental diversity [26], we should not be surprised to find many such examples among cnidarians.

## Results

### The *Acropora Sox *gene complement

**Figure 1:**
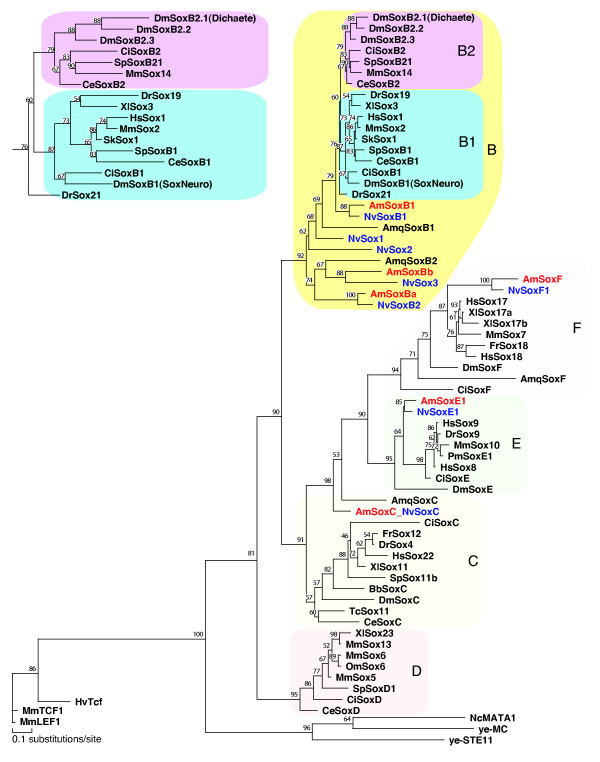
**Maximum Likelihood phylogenetic analyses of *Acropora Sox *genes in MolPhy version 2.3**[49]** using the Dayhoff model of protein evolution and local rearrangement of the NJ trees (1,000 bootstraps). ***Acropora millepora Sox *genes are shown in red, while *Nematostella vectensis *genes are shown in blue. The inset, showing the B1 and B2 families, has been stretched horizontally to clarify the relationship between the B1 and B2 genes for the reader. Species names are abbreviated as follows; Am, coral, *Acropora millepora*; Amq, demosponge, *Amphimedon queenslandica*; Bb, Japanese lancelet, *Branchiostoma belcheri*; Ce, nematode, *Caenorhabditis elegans*; Ci, ascidian, *Ciona intestinalis*; Dm, fruit-fly, *Drosophila melanogaster*; Dr, zebrafish, *Danio rerio*. Fr, Japanese pufferfish, *Fugu rubripes*; Gg, chicken, *Gallus gallus*; Hs, human, *Homo sapiens*; Hv, hydra, *Hydra vulgaris*; Mm, mouse, *Mus musculus*; Nc, red bread mold, *Neurospora crassa*; Nv, sea anemone, *Nematostella vectensis*; Om, rainbow trout, *Oncorhynchus mykiss*; Pm, sea lamprey, *Petromyzon marinus*; Sk, hemichordate, *Saccoglossus Kowalevskii*; Sp, sea urchin, *Strongylocentrotus purpuratus*; Tc, red flour beetle, *Tribolium castaneum*; Xl, frog, *Xenopus laevis*; ye-, yeast, *Schizosaccharomyces pombe*.

Full-length cDNAs encoding two Sox proteins (AmSoxBb and AmSoxB1) were identified during the course of an ongoing *Acropora *EST project [27,28]. Four other *Acropora Sox *genes were identified by a PCR-based approach. Redundant PCR primers (designed from an alignment of HMG domains of a range of Sox proteins) allowed the generation of four novel *Acropora *PCR products, and for each of these, full-length cDNAs were isolated by library screening. Based on domain structure and phylogenetic analyses, these six *Sox *genes were classified as *AmSoxB1 *(Genbank # EU784831, Additional File 1), *AmSoxBa *(Genbank # EU784832, Additional File 2), *AmSoxBb *(Genbank # EU784833, Additional File 3), *AmSoxC *(Genbank # EU784834, Additional File 4), *AmSoxE1 *(Genbank # EU784835, Additional File 5) and *AmSoxF *(Genbank # EU784836, Additional File 6). Each of these genes has a clear *Nematostella *homolog (Fig. 1 and Additional Files 1, 2, 3, 4, 5, 6); *AmSoxBa *is orthologous with *NvSoxB2*, and *AmSoxBb *with *NvSox3*.

Phylogenetic analysis based on the HMG domain (Fig. 1) resolved bilaterian Sox proteins into Types B-F, supporting previous analyses (e.g. [2]), and four of these five major Sox groups were strongly supported (> 90% bootstrap support). Five of the six *Acropora *Sox proteins fell into these clades, the sole exception being *Acropora *SoxC, which is discussed below; moreover the clade consisting of bilaterian SoxC proteins has only moderate (57%) bootstrap support. Although *Acropora *has *Sox *genes that are clearly of the *SoxB *type (Fig. 1), the relationship of the non-Bilaterian *SoxB *genes with the bilaterian *SoxB1 *and *SoxB2 *clades is not simple. The analysis shown implies that the bilaterian *SoxB2 *type arose after the cnidarian/bilaterian split (Fig. 1, inset). One of the *Acropora *SoxB proteins, AmSoxB1, and its likely *Nematostella *ortholog (NvSoxB1) form a sister clade to all of the bilaterian SoxB sequences. To avoid confusion with bilaterian SoxB2 orthologs, we refer to the *Acropora *ortholog of *Nematostella *"*NvSoxB2*" as *AmSoxBa*. The HMG domains encoded by *AmSoxBa *and *AmSoxBb*, as well as their *Nematostella *orthologs (*NvSoxB2 *and *NvSox3*), carry a single amino acid (K or R, respectively) residue insertion (at position 75 in Additional File 7) and, together with the sponge gene *AmqSOXB2*, these four anthozoan sequences form a sister clade to all of the other SoxB proteins. Inclusion of the full complement of human Sox HMG domains (Additional File 8) does not alter the implied relationships between sequences from non-Bilateria, but decreases support for some nodes within the tree.

**Figure 2:**
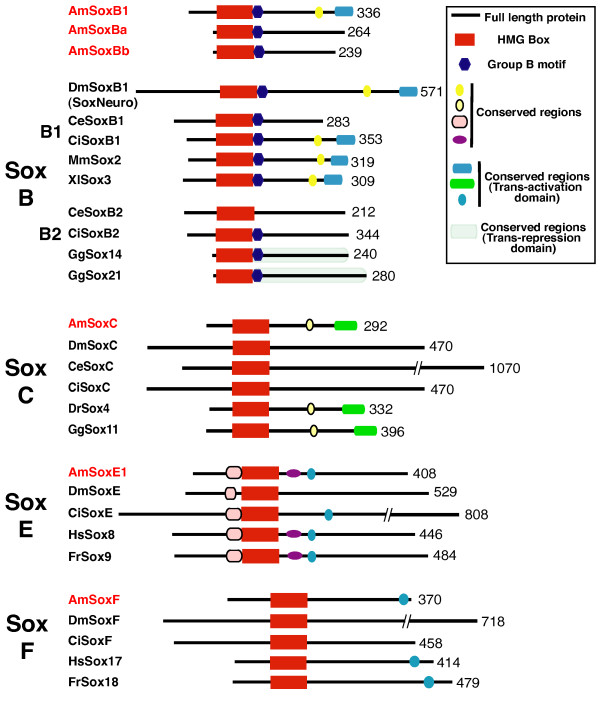
**Schematic drawings, not to scale, comparing *Acropora *Sox proteins with those of various bilaterians, across the main Sox families (B-F).** The conserved motifs compared are identified in the inset. Numbers of amino acids in the full length proteins are indicated on the right. Species names are abbreviated as follows; Am, coral, *Acropora millepora*; Ce, nematode, *Caenorhabditis elegans*; Ci, ascidian, *Ciona intestinalis*; Dm, fruit-fly, *Drosophila melanogaster*; Dr, zebrafish, *Danio rerio*. Fr, Japanese pufferfish, *Fugu rubripes*; Gg, chicken, *Gallus gallus*; Hs, human, *Homo sapiens*; Mm, mouse, *Mus musculus*; Xl, frog, *Xenopus laevis*.

To better understand evolutionary relationships of the *Acropora *Sox repertoire, the domain structures of the predicted *Acropora *Sox proteins were compared with their counterparts from chordates and protostome invertebrates (Fig. 2, Additional Files 1, 2, 3, 4, 5, 6). In these comparisons, the similarities between the *Acropora *Sox proteins and their vertebrate homologs are striking, whereas the members of these Sox families from invertebrates (fly, nematode, ascidian) are often less similar at the level of domain structure; see, for example, the *Acropora *members of the SoxE and SoxF groups (Fig. 2). For group E, a conserved region located just N-terminal of the HMG domain (Fig. 2) is observed in AmSoxE1 and several conserved regions are located just C-terminal of the HMG domain of AmSoxE1. The conserved short motif EF(D/E)QYL in the C-terminal region of group F genes is essential for transcriptional activation [29]. This motif is completely conserved in AmSoxE1 and AmSoxF proteins (Fig. 2, Additional Files 5 and 6), suggesting that AmSoxE1 and AmSoxF may also function as transcriptional activators.

The *Acropora *group B Sox proteins (AmSoxBa, AmSoxBb and AmSoxB1) each contain a group B-specific motif located immediately C-terminal of the HMG domain (Fig. 2 SoxB, Additional Files 1, 2, 3). In addition, the AmSoxB1 protein contains two other domains characteristic of the SoxB1 sub-type (Fig. 2 SoxB), one of which has been shown to have transcriptional activation properties [30], implying that AmSoxB1 may also be able to activate transcription.

Despite not being within the SoxC clade in the phylogenetic analysis of HMG box sequences (Fig. 1), the AmSoxC protein is strikingly similar to vertebrate SoxC proteins in terms of its overall domain structure (Fig. 2 SoxC, Additional File 4). In addition to the HMG box, two other motifs that are characteristic of vertebrate SoxC proteins are also present in the C-terminal region of the AmSoxC protein; one of these has a predicted transactivation function [2,31], consistent with a function in a transcriptional activation for AmSoxC. Note that these domains could not be identified in a wide range of other invertebrate SoxC proteins. The overall structural similarities between AmSoxC and vertebrate SoxC proteins support the classification of the former as a member of Sox group C, despite only moderate support from phylogenetic analysis of the HMG domain (Fig. 1).

### *Acropora SoxB *and *SoxE *genes are expressed in the presumptive ectoderm and endoderm, respectively, during gastrulation

**Figure 3:**
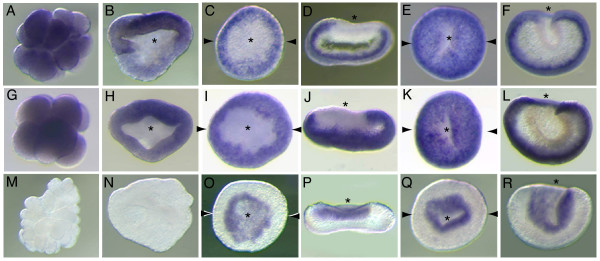
**Spatial expression patterns of *AmSoxBb*, *AmSoxB1 *and *AmSoxE1 *during early embryogenesis: A-F) *AmSoxB1*; (G-L) *AmSoxBb*; (M-R) *AmSoxE1*.** (A, G, M) Early cleavage stage. (B, H, M) Blastula (prawnchip) stage. (C, I, O) Early donut stage, during gastrulation. (D, J, P) Transverse sections of C, I, O, respectively. (E, K, Q) Late donut stage, finishing gastrulation. (F, L, R) Transverse sections of E, K, Q respectively. Asterisks indicate the blastopore. Paired arrows on a panel of the figure indicate that the next panel is a section in the plane of the arrows (e.g. D is a transverse section of the embryo shown in C). The speed of embryonic development is temperature dependent, so we have not attempted to give ages of the embryonic stages in this and later figures. Typical ages for the various stages are available in Figure 2 of Ball et al. [38].

The spatial expression patterns of *AmSoxBb *and *AmSoxB1 *during early embryogenesis are very similar. Maternal *AmSoxBb *and *AmSoxB1 *transcripts are uniformly distributed in the unfertilized egg (not shown), and early cleaving (Fig. 3A, G), and "prawn-chip" embryos (not shown). At the initiation of gastrulation (late "prawn-chip" stage), *AmSoxBb *and *AmSoxB1 *transcripts become depleted in the presumptive endoderm (Fig. 3B, H). In early gastrulae (early "donut" stage; 20 h), the *SoxB *transcripts are clearly restricted to the presumptive ectoderm (Fig. 3C, D, I, J) and this restriction is maintained until gastrulation is complete (Fig. 3E, K). By contrast, *AmSoxE1 *transcripts cannot be detected in unfertilized eggs or early cleaving embryos, implying that *AmSoxE1 *transcripts are not provided maternally (Fig. 3M). *AmSoxE1 *transcripts are first detected in the presumptive endoderm at the early "donut" stage (Fig. 3O). During gastrulation, *AmSoxE1 *expression is maintained specifically in the presumptive endoderm (Fig. 3Q), thus throughout gastrulation the *SoxB *and *SoxE *genes specifically mark the presumptive ectoderm and endoderm respectively. *AmSoxE1 *expression remains endodermal throughout larval and early post-settlement development (data not shown).

### *AmSoxC *is expressed in putative sensory neurons

**Figure 4:**
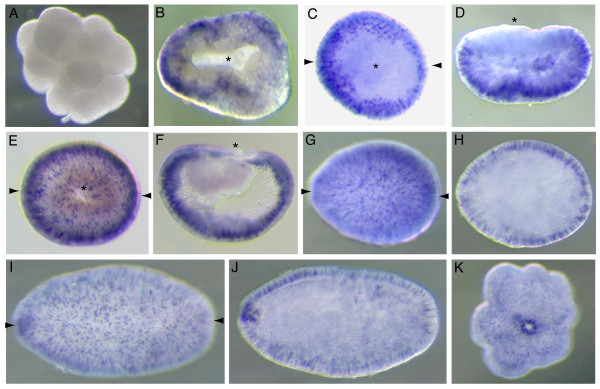
***AmSoxC *****shows cell specific expression in the ectoderm from early embryogenesis through post-settlement: **(A) Early cleavage stage. (B) Prawnchip stage. (C) Initiation of gastrulation at the early donut stage. (D) Transverse section of C. (E) Donut stage embryo, during gastrulation. (F) Longitudinal section of E. (G) Pear stage. (H) Longitudinal section of G showing that expression is restricted to the ectoderm. (I) Planula stage. (J) Longitudinal section of I. The cell specific expression in the ectoderm continues and oral pore staining is observed. (K) Post settlement polyp. Though the ectodermal cell specific expression becomes weaker, the oral pore staining is still maintained. Paired arrows on a panel of the figure indicate that the next panel is a section in the plane of the arrows (e.g. D is a transverse section of the embryo shown in C).

*AmSoxC *mRNA was not detected in unfertilized eggs or early cleaving embryos (Fig. 4A); transcripts were first detected at the "prawn-chip" stage. *AmSoxC *is initially expressed in scattered cells in the presumptive ectoderm (Fig. 4B). Slightly later, transcription appears be restricted to a more uniformly distributed subset of cells throughout the presumptive ectoderm during gastrulation. This cell-specific expression first becomes apparent in the early donut stage (Fig. 4C, D; 20 h). Expression is restricted to individual ectodermal cells throughout gastrulation (Fig. 4E, F), and this pattern is maintained throughout larval development (Fig. 4G–J) and continues after settlement. At the planula stage, in addition to the cell specific expression, more generalized *AmSoxC *expression is detected in the outer ectoderm around the oral pore (Fig. 4I, J). In the post-settlement stage, although the expression of *AmSoxC *in the oral pore is still maintained, the number of *AmSoxC *expressing cells decreases (Fig. 4K). The significance of the oral expression is unclear.

**Figure 5:**
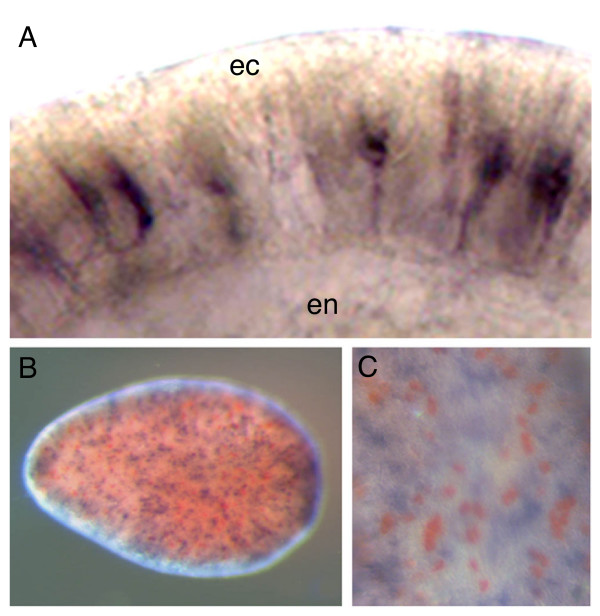
**Cell morphology of *AmSoxC *expressing cells.** (A) High magnification view of the ectoderm of in situ stained embryos (pear stage). (B) Double in situ hybridization of *AmSoxC *and *Amlipase *(planula stage). *AmSoxC *is stained black and *Amlipase *is red. (C) High magnification view of the surface of B. No overlapping staining is observed. Abbreviations: en = endoderm, ec = ectoderm.

At higher magnification (Fig. 5), the morphologies of the ectodermal cells expressing *AmSoxC *are revealed. These cells are thin and bipolar with the cell body located midway across the ectoderm (Fig. 5A) and cytoplasmic projections extending to both of its borders. In some cases, *AmSoxC *expressing cells are connected by thin cytoplasmic projections (Fig. 5A). The morphology of some of these *AmSoxC *expressing cells is consistent with that of 'type 1' sensory neurons, one of two presumed neuron classes identified by immunostaining with antibodies directed against the cnidarian neuropeptide RFamide [32,33].

One way of testing the prediction that *AmSoxC *is expressed in neurons is to compare its expression pattern with that of other genes that are unlikely to be expressed there. One such highly expressed gene is *Amlipase*, which codes for an enzyme unlikely to be abundant in neurons but likely to be present in gland cells, which form an important component of the ectoderm. To further investigate the identity of the *AmSoxC *expressing cells double *in situ *hybridization studies were carried out using *AmSoxC *and *Amlipase *(Fig. 5B, C); cells expressing *Amlipase *were visualised using Sigma Fast Red (red) and those expressing *AmSoxC *stained using NBT/BCIP (purple/black). As can be seen in Fig. 5B, C, the two probes stain mutually exclusive cell populations, indicating that *Amlipase *and *AmSoxC *are not co-expressed. These expression data are consistent with the hypothesis that the *AmSoxC*-expressing cells may constitute a neural cell type.

**Figure 6:**
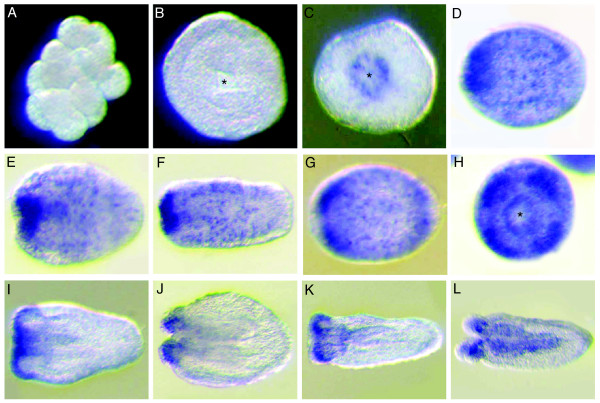
**Spatial expression pattern of *NvSoxC *during *Nematostella *development.** Asterisks indicate the blastopore (B, C) or oral pore (H) when it faces out of the page, otherwise the oral pore is oriented to the left and aboral is to the right. (A) Early cleavage stage, (B) gastrula stage, (C) late gastrula stage, immediately after blastopore closure. (D-F) planula stage, (G) expression in pre-tentacles, (H) oral pore view of G, (I) early metamorphosing planula, (J) late metamorphosing planula stage with further retraction of pharynx towards aboral pole, (K) late metamorphosing planula which has finished elongating, (L) primary polyp.

*Nematostella *has a clearly orthologous gene (*NvSoxC*; Fig. 1) whose expression pattern has not previously been reported. This gene is expressed in a broadly similar pattern to its *Acropora *counterpart throughout development (Fig. 6), but some differences were apparent. For example, in contrast to the situation in *Acropora*, *Nematostella NvSoxC *transcripts were not detected at the blastula or gastrula stages, but were first detected at the late gastrula/very early planula stage, in the margin of the oral pore. At the planula stage, in addition to the maintenance of the early (oral pore margin) expression pattern, *NvSoxC *is expressed in individual cells in the ectoderm (Fig. 6D–F). As development progresses, the expression surrounding the oral pore is altered to where the tentacle buds are initiated (Fig. 6G, H), though the ectodermal cell specific expression is still maintained. In the metamorphosing planula, the ectodermal cell specific expression is no longer detected (Fig. 6I). In the primary polyp, the expression in the tentacle buds persists and *NvSoxC *expression is detected in the ectoderm of the pharynx (Fig. 6J–L).

### Expression analysis of *AmSoxBa *and *AmSoxF*

**Figure 7:**
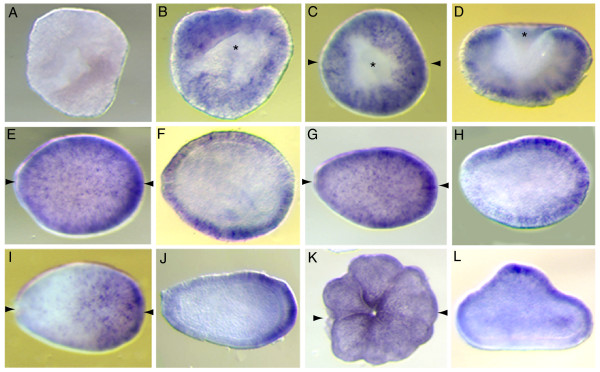
**Spatial expression pattern of AmSoxBa during development. **(A) There is no expression in the early prawnchip stage, (B) Initiation of gastrulation in the late prawnchip stage. AmSoxB2 transcripts appear in the presumptive ectoderm with some cell specific staining. (C) At the donut stage the general ectodermal expression and some cell specific staining persist. (D) Transverse section of C. Expression is restricted to the presumptive ectoderm. (E) Pear stage (F) Longitudinal section of E shows ectodermal expression. (G) Late pear or early planula stage. (H) Longitudinal section of G. (I) Planula stage (96 h). Cell specific expression is restricted to the aboral half of the ectoderm. (J) Section image of I. (K) Post settlement polyp. (L) Transverse section of K. Expression is now missing from the aboral ectoderm. Asterisks indicate the position of the blastopore. (E-J) Oral pore is oriented to the left and aboral side to the right. Paired arrows on a panel of the figure indicate that the next panel is a section in the plane of the arrows (e.g. D is a transverse section of the embryo shown in C).

Unlike the other *Acropora *B type *Sox *genes, *AmSoxBa *transcripts could not be detected in unfertilized eggs, or in early cleaving and prawn-chip stage embryos, indicating that *AmSoxBa *is not a maternal transcript (Fig. 7A). Expression of *AmSoxBa *is first detected at the late prawnchip stage (Fig. 7B), which corresponds to the time at which gastrulation begins. *AmSoxBa *transcripts appear in the presumptive ectoderm with some cell specific staining. During gastrulation, the general ectodermal expression and some cell specific staining persist (Fig. 7C). After completion of gastrulation, *AmSoxBa *exhibits cell specific ectodermal expression in the pear and planula stages (Fig. 7E–H). The nuclei of *AmSoxBa *expressing cells are located centrally or closer to the surface of the ectoderm. The cells are thin with several projections extending from the core region. In the late planula stage, cell specific expression is restricted to the aboral ectoderm (Fig. 7I, J). After metamorphosis, *AmSoxBa *is expressed throughout the entire ectoderm (Fig. 7K, L) except in the regions around the polyp base (Fig. 7L).

**Figure 8:**
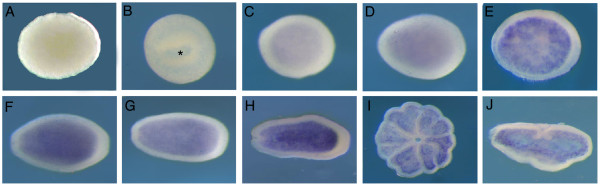
**Spatial expression pattern of AmSoxF throughout development.** An asterisk indicates the blastopore. (C-H) Oral pore is oriented to the left and aboral side to the right. (A) Unfertilized egg. (B) late donut stage (C-H) weak endodermal expression appears at the pre-pear stage, strengthening throughout the late pear and planula stages. (I-J) Endodermal expression continues post-settlement.

*AmSoxF *transcripts could not be detected in unfertilized eggs, in early cleaving embryos (Fig. 8A), prawnchip or donut stages (during gastrulation; Fig. 8B), consistent with RT-PCR (not shown). *AmSoxF *expression is first detectable at the pre-pear stage (Fig. 8C, D); during the pear stage, *AmSoxF *is expressed throughout the entire endoderm (Fig. 8E), and this pattern persists until after settlement (Fig. 8F–J).

## Discussion

### Comparison of the *Acropora *and *Nematostella *Sox complements

Each of the six *Acropora Sox *genes reported has a clear counterpart in *Nematostella*, and these pairs of genes are probable orthologs. A total of 14 Sox sequences has been reported from *Nematostella *[9], so presumably more *Acropora *genes will be discovered as a result of continuing transcript characterisation using 454 sequencing. However, most of the *Nematostella *genes without *Acropora *counterparts (*NvSoxA*, *NvSoxE2*, *NvSoxF2*, *NvSoxJ*, *NvSox4*, *NvSox5*) were predicted from genomic sequence data so their existence as functional transcripts remains unproven. In our analyses (Fig. 1), three of the *Nematostella Sox *genes (*NvSox1*, *NvSox2*, *NvSox3*) that were unassigned in the Magie et al. study [9] fell clearly within the SoxB clade. Factors contributing to these differences, and the significantly higher resolution in our analysis, include the deliberate exclusion of highly divergent bilaterian sequences and the use of only complete HMG domain sequences in our case.

### Anthozoan *Sox *genes and the evolutionary divergence of the Sox family

*Sox *genes have previously been reported from both cnidarians and sponges [6-9] but, as a number of these surveys have been based on PCR screens or scanning genomic resources, pseudogenes may have been included and in some cases assignments have been based on incomplete HMG domain sequences. The analyses presented here are based on complete HMG domain data derived from cDNAs, overcoming previous limitations. In terms of the representation of the ten (A-J) recognized classes of *Sox *genes, our studies confirm the presence of group B, C, E and F Sox types in cnidarians, and are consistent with the absence of group D, G, H, and I as reported by Magie et al. [9]. However, in our analyses *Nematostella *genes previously assigned to groups A and J fell into group B (Additional File 9) in agreement with [6]. The strong bootstrap support (≥ 90%) for monophyly of Sox groups B, E and F in our analyses (Fig. 1) indicates that each of these (and probably also the SoxC type) were distinct in the common ancestor of cnidarians and bilaterians, and the assignment of sponge genes to the Sox B, F, and C classes in our analyses implies that these classes had already diverged in Urmetazoa (the common ancestor of all animals), consistent with [6]. Hence our analysis is broadly consistent with that in a recent study of the *Sox *gene complement of the sponge *Amphimedon *[8].

According to our analyses none of the cnidarian Sox sequences fall into subgroup B2, the distinctness of which is well supported (79%) by bootstrap probability, and the relationship of the non-bilaterian sequences to subgroup B1 is not simple (Fig. 1). It appears that Sox group B2 of the Bilateria may have arisen from a B1-like precursor after the Cnidaria/Bilateria divergence. However, the extent to which the phylogeny might be biased by the presence of an extra amino acid residue in two of the *Acropora *HMG domains (and their *Nematostella *counterparts) is not yet clear. Therefore, although the tree presented here (Fig. 1) contradicts the conclusions of Larroux et al. [8] with respect to divergence within the Sox B class, relationships between the SoxB1 and SoxB2 types remain unresolved. The additional amino acid residue in the HMG domains of two *Acropora *Sox proteins and their likely *Nematostella *orthologs, a lysine residue in the case of AmSoxBa and NvSoxB2 and an arginine residue in AmSoxBb and NvSox3, is thus far unique to these group B genes, and its origin may post-date the Cnidaria/Bilateria split.

In terms of HMG domain sequences, sizes and protein domain structures, the *Acropora *Sox proteins are strikingly similar to the vertebrate members of each of these Sox groups, whereas more differences are apparent in vertebrate/*Drosophila *and vertebrate/*Caenorhabditis *comparisons. This implies that, as is the case for some other genes [27], vertebrate and cnidarian *Sox *genes may more closely reflect ancestral characteristics than do their fly and worm counterparts. However, in some respects, the structures of the sponge Sox proteins [7] differ substantially from their cnidarian and bilaterian counterparts. The sponge sequences are more divergent in the HMG domains, lack other obvious conserved domains, and differ in overall size and position of the HMG domain. It is unclear, however, whether these differences reflect divergence within the sponge lineage or innovations within the cnidarian/bilaterian lineage potentially underpinning the transition to tissue level organization.

### Expression patterns of *Acropora Sox *genes – glimpses of ancestral functions?

The restriction of the *Acropora SoxBb *and *SoxB1 *mRNAs to the presumptive ectoderm and *SoxE1 *to the presumptive endoderm during gastrulation is suggestive of roles for these genes in germ-layer specification. Although often referred to as neural markers [4], members of Sox group B are also important for germ layer formation and gastrulation in both vertebrates and invertebrates. *Sox3 *(a group B1 gene) regulates gastrulation and germ layer formation in both *Xenopus *and zebrafish [22]. In the sea urchin, *SoxB1 *and *B2 *are expressed in the presumptive ectoderm during gastrulation and are necessary for gastrulation and vegetal development [23]. In the hemichordate *Saccoglossus kowalevskii*, *Sox1/2/3 *(a *SoxB *gene) is expressed in the entire ectoderm of the gastrula embryo [19]. These similarities suggest an ancestral function of group B *Sox *genes in germ layer specification. The significance of the *AmSoxE1 *expression pattern is more difficult to assess, as few early *SoxE *expression patterns have been reported. Roles for *Sox9 *(group E) genes in neural crest development [34-36] presumably reflect co-option.

Zygotic *AmSoxE1 *expression starts from the late prawnchip or early donut stage, and this gene marks the presumptive endoderm during gastrulation (Fig.3). Although this is suggestive of a role for *AmSoxE1 *in endoderm determination, there are no clear precedents for this. In the sea urchin, *SoxE *transcripts are localized in small micromere descendents at the tip of the archenteron during gastrulation [37], but this gene is not expressed in the blastula stage. However, few comparative expression patterns have been reported, and more general roles for *SoxE *genes in early tissue patterning cannot yet be ruled out. Although *AmSoxF *is not expressed until after gastrulation (Fig. 8), expression is limited to the endoderm, so it is possible that *AmSoxF *plays a role in the maintenance of endodermal identity.

### Heterogeneity in early *Sox *expression patterns within the Anthozoa

Although *Acropora *and *Nematostella *are both members of the same subclass (Hexacorallia, or Zoantharia) within the cnidarian Class Anthozoa there are apparent differences in the expression patterns of presumably orthologous genes. Some of these differences may just reflect the more complete series of early developmental time points reported for *Acropora*, while others presumably result from fundamental differences in the overall developmental biology of the anemone and the coral (see [38,39]).

The early development of *Nematostella *is now well documented up to blastopore closure, although some details of the mechanism of gastrulation remain equivocal [9,40-42]. Later development is less well understood with the most complete description still being that of Hand and Uhlinger [43]. *Acropora *development has been characterised much less thoroughly although it is clear that there are some dramatic differences between the two species [44,45]. Firstly, it appears that the *Acropora *egg contains much more yolk than that of *Nematostella*, consistent with the frequently longer planktonic life of the former species. Secondly, the prawn-chip stage is more exaggerated in *Acropora*, in that many more cells are present at this stage, so that the embryo takes on the appearance of a warped dinner plate, rather than a small bowl. The morphology of the post-gastrulation larva in the two species is also quite different due to the large amount of yolk present in *Acropora*. Thus the *Nematostella *planula has a far more developed endoderm and structures such as the septa are apparent from shortly after blastopore closure. In contrast, the early *Acropora *planula has a poorly developed endoderm consisting of a thin layer of cells lying beneath the mesogloea plus small cells scattered among the large yolk cells that pack the cylindrical central axis of the larva. It is only late in planula development that the tightly packed core of yolk begins to thin at the oral end and septal development becomes apparent. These morphological differences mean that even when a gene is functioning in a similar manner its pattern of expression may appear somewhat different.

Magie et al. [9] comment that *NvSox3 *is the only *Nematostella Sox *gene that is highly expressed maternally. Its *Acropora *ortholog, *AmSoxBb *(Fig. 3G–L) is also expressed in the egg and in early stages of embryonic cell division. A second *Acropora *gene, *AmSoxB1*, is also maternal, as evidenced by detection of the mRNA from the earliest stages of development (Fig. 3A), and has a very similar pattern of expression to *AmSoxBb*. The earliest stage at which expression of *NvSoxB1 *(the ortholog of *AmSoxB1*) is shown by Magie et al. [9] is the gastrula, when its mRNA is restricted to the aboral ectoderm and a discrete area around the blastopore which will give rise to the pharynx. *Sox E *may be an example of the phenomenon noted above, where orthologs may function similarly in spite of initial expression patterns that appear quite different. Thus, early expression in *Nematostella *appears to be ectodermal while in *Acropora *it is clear that the expression will clearly become endodermal much earlier. However, by the end of gastrulation the expression is endodermal in both organisms so the way this expression is arrived at may not be functionally significant.

The cell-specific expression pattern of *NvSoxC*, the *Nematostella *ortholog of *AmSoxC*, in the ectoderm is broadly similar to that of the *Acropora *gene. However, there are obvious differences. Firstly, expression begins in *Acropora *well before the blastopore has closed while expression of *NvSoxC *begins later – expression of the anemone gene could not be detected during gastrulation. A second difference is that expression associated with the oral pore appears just after the blastopore has closed in *Nematostella *(Fig. 6), while in *Acropora *this is not seen prior to the planula stage. Finally, while *NvSoxC *is clearly expressed in developing tentacles in *Nematostella *(Fig. 6J–L), no such expression is seen in *Acropora *at comparable (i.e. later in development) stages of tentacular development (not shown).

The expression pattern of *NvSoxC *reported here (Fig. 6) has some striking similarities with that previously reported for another *Nematostella Sox *gene, the B type gene *NvSox2 *[9]. Like *NvSoxC*, *NvSox2 *is expressed in a subset of ectodermal cells that are distributed in a scattered pattern early in post-gastrulation development but become restricted to the ends of the developing tentacles later; the similarity is most striking at the stage shown as Fig. 6H – compare this pattern for *NvSoxC *with Fig. 3ll in Magie et al. [9] for *NvSox2*. Unfortunately, direct comparisons are limited by the fact that no *NvSox2 *in situ data are available for the period corresponding to Fig. 6D–G. Note that the two *Nematostella *genes are on separate scaffolds in genome assembly v1.0 (JGI) so it is unlikely that they are tightly linked.

Direct comparisons of expression patterns later in development are complicated by the more extensive differences between *Nematostella *and *Acropora *(e.g. the incomplete metamorphosis of the anemone). Whilst *AmSoxBa *and *NvSoxB2 *have similar cell-specific expression patterns throughout gastrulation, in *Acropora *this ectodermal pattern persists through to the planula stage (Fig. 7), whereas *NvSoxB2 *is expressed in a cell-specific manner in both endoderm and ectoderm [7]. Immediately prior to settlement, expression of *AmSoxBa *becomes restricted to the aboral half of the ectoderm (Fig. 7), but nothing like this axial restriction is seen during *Nematostella *development before tentacle formation.

## Conclusion

The short and manipulable [43,46] life cycle of *Nematostella*, as well as its position as the first cnidarian with a fully sequenced genome, have led it to prominence as a representative of the large and diverse phylum Cnidaria. Because *Nematostella *appears to have retained much of the ancestral complexity of some gene classes (for example, the TGFb and Wnt signaling molecules; [28,47]), there is an assumption that by comparison of its gene repertoire and expression patterns with higher animals ancestral characteristics can be inferred. However, *Nematostella *has apparently also undergone significant gene loss in some gene families – extensive losses in the case of the Fox transcription factor family [25] – and even between *Acropora *and *Nematostella*, both members of the anthozoan subclass Hexacorallia (Zoantharia), the expression patterns of orthologous genes appear to differ significantly. Whilst these latter differences may often simply reflect divergent patterns of development, they underscore the need for comparative data for other cnidarians – ancestral characteristics can be inferred with much greater confidence when the inference is based on multiple representatives. Given their diversity of developmental programs, this principle is likely to be particularly significant in the case of phylum Cnidaria.

## Methods

### Animal sampling

Eggs and embryos of *Acropora millepora *were collected at Magnetic Island (Latitude 19°09' South; Longitude 146°49' East) and Orpheus Island (Latitude 18°28' South; Longitude 146°25' East) during the coral mass-spawning events of 2004, 2005 and 2006.

### Isolation of *Sox *genes from *Acropora millepora*

Two *Sox *genes (*AmSoxBb *and *AmSoxB1*) were isolated during the *Acropora millepora *EST projects [27,28]. In order to search for more *Sox *genes, RT-PCR amplification of the highly conserved HMG domain of *Sox *genes from *Acropora *was attempted. First strand cDNAs from different embryonic stages were used as an RT-PCR template. Degenerate primers were designed based on highly conserved regions of the HMG domain. Primer sequences were; SoxFw (5'-CCNATGAAYGCNTTYATNGTNTGG-3') and SoxRv (5'-GGNYKRTAYTTRTART-YNGG-3'), corresponding to the amino acid sequences PMNAF(M/I)VW and P(N/D)YKY(Q/R/K)P. PCR conditions were as follows: 94°C for 30 second, 42°C for 30 second, 72°C for 30 second (5 cycles) and then 94°C for 30 second, 50°C for 30 second, 72°C for 30 second (35 cycles). DNA fragments of the expected size (208 bp) were cloned into pGEM-T vector (Promega) and sequenced either using the ET Dynamic sequencing kit (Amersham Biosciences) or by Macrogen Inc. (Seoul, South Korea). Novel *Sox *gene fragments were subsequently used as probes for cDNA library screening. The l-UniZap XR *A. millepora *cDNA library was constructed using messenger RNA from adult coral tips. cDNA library screening was carried out as described in Miller et al. [48].

### Phylogenetic analysis

In the case of the vertebrate sox proteins, relatedness in the HMG domain is a good indicator of overall relatedness [2]. Full length HMG domain sequences (79 amino acids) encoded by the *Sox *genes of *Acropora *and other animals were aligned by ClustalW prior to Maximum Likelihood phylogenetic analyses in MOLPHY version 2.3 [49] using the Dayhoff model of protein evolution and local rearrangement of the Neighbor-joining tree. In the case of non-bilaterian *Sox *genes, only those for which cDNAs encoding complete HMG domains have been identified were used for phylogenetic analysis [6,9]. Genes have been named consistent with [2]. Where other names are in common use they are indicated in brackets [e.g. DmSoxB2-1 (Dichaete)]. The resulting tree was rooted using established outgroups; red bread mold *Neurospora crassa *MATA1, yeast *Schizosaccharomyces pombe *MC, STE11, *Mus musculus *TCF1, LEF1 [50], and *Hydra vulgaris *Tcf [51].

### Fixation and whole mount *in situ *hybridization of coral embryos

*Acropora millepora *embryos were processed as described by Anctil et al. [52] up to BCIP/NBT color development. Color development was then stopped by washing several times with PBT, and the backgrounds cleared in ethanol.

For double *in situ *hybridization of *AmSoxC *and the *Acropora lipase *gene (*Amlipase*; Genbank # EU770585) the *Amlipase *gene was labeled with fluorescein using Fluorescein Labeling Mix (Roche). Because the expression of *AmSoxC *is much weaker than that of *Amlipase*, embryos were incubated with *AmSoxC *DIG-probe overnight, and then fluorescein labeled *Amlipase *probe was added to the hybridization solution with hybridization continuing for 24 hours after addition. The DIG-labeled *AmSoxC *cRNA probe was detected using a 1:1500 dilution of anti-DIG antibody followed by development in BCIP/NBT and background removal by washing in ethanol. The AP-conjugated anti-DIG-antibody was then fully removed by washing with 100 mM glycine·HCl, pH2.2, 10 minutes at room temperature, followed by several washes in PBT. Finally fluorescein-labeled *Amlipase *probe was detected using a 1:800 dilution of AP-conjugated anti-fluorescein antibody (Roche) followed by development with Sigma Fast Red (Sigma). The stained embryos were mounted in 80% glycerol and kept at 4°C. Images were captured with a SPOT digital camera and processed using Adobe Photoshop.

### Whole mount in situ hybridization of *Nematostella vectensis*

For synthesis of the *NvSoxC *DIG-labeled RNA probe, the *NvSoxC *nucleotide fragment was amplified by PCR from a *Nematostella vectensis *genomic library. Primers for *NvSoxC *were designed based on the predicted ORF nucleotide sequence. Primer sequences were as follows: NvSoxC-Fw, 5'-CTAGTGATGATGATGGTGCTA-CAG-3', NvSoxC-Rv, 5'-CAGGCTGACCGTATCGAG-3'. PCR conditions were as follows: 94°C for 30 seconds, 50°C for 30 seconds, 72°C for 30 seconds (35 cycles). DNA fragments of the expected size (981 bp) were cloned into the pGEM-T Easy Vector (Promega). The plasmid template was linearised by cutting with *Nco*I, and SP6 RNA polymerase used for RNA probe synthesis. The published *in situ *hybridization protocol [53] was followed with the following modifications. Fixed animals were rehydrated from 70% ethanol, treated with 20 μg/ml proteinase K for 15 min, washed repeatedly in PBT, and then refixed in 4% paraformaldehyde for 20 min. Following several PBT washes, the embryos were transferred into hybridization solution and treated as described above for *Acropora*. Hybridization was at 42°C for 72 h. Free probe was removed by several washes in hybridization wash solution at 50°C. Probe detection, color development and image capture were as described above. Determination of *Nematostella *developmental stages was based on Hand and Uhlinger [43].

## Authors' contributions

CS carried out the molecular genetic analyses and drafted the manuscript. AI, UT and DH contributed to the molecular analyses and sequence alignment, and commented on drafts of the manuscript. DM did the phylogenetic analyses, and EB and DM conceived the study, were responsible for the cellular and evolutionary interpretation of the data, and contributed substantially to writing the manuscript. All authors read and approved the final manuscript.

## Supplementary Material

Additional file 1 **Sequence analysis of *AmSoxB1*.** (A) The nucleotide sequence and deduced amino acid sequence of the *AmSoxB1 *cDNA. The 1888 bp *AmSoxB1 *cDNA contains an open reading frame (ORF) of 1008 bp, corresponding to 336 amino acids. An asterisk indicates the stop codon. The 79 amino acids of HMG box sequence are highlighted in red. Numbers on the left side represent the nucleotide sequence; numbers on right side represent the amino acid sequence. A putative polyadenylation site is underlined. (B) Boxshade alignment of *AmSoxB1 *and other subgroup B1 *Sox *genes. The HMG domain is underlined in red. The group B motif is underlined in blue. Asterisks indicate the key residues of group B. Highly conserved regions (i and ii) are underlined. The species names are abbreviated as follows; Am, coral, *Acropora millepora*; Ce, nematode, *Caenorhabditis elegans*; Ci, ascidian, *Ciona intestinalis*; Dm, fruit-fly, *Drosophila melanogaster*; Mm, mouse, *Mus musculus*; Nv, sea anemone, *Nematostella vectensis*; Sk, hemichordate, *Saccoglossus kowalevskii*; Sp, sea urchin, *Strongylocentrotus purpuratus*; Xl, frog, *Xenopus laevis*. Click here for file

Additional file 2 **Sequence analysis of *AmSoxBa*.** (A) The nucleotide sequence and deduced amino acid sequence of the *AmSoxBa *cDNA. The 79 amino acids of HMG box sequence are highlighted in red. Numbers on the left side represent the nucleotide sequence; numbers on right side represent the amino acid sequence. (B) Boxshade alignment of *AmSoxBa *and other subgroup B2 *Sox *genes. The HMG domain is underlined in red. The group B motif is underlined in blue. Asterisks indicate the key residues of group B. The species names are abbreviated as follows; Am, coral, *Acropora millepora*; Ce, nematode, *Caenorhabditis elegans*; Ci, ascidian, *Ciona intestinalis*; Dm, fruit-fly, *Drosophila melanogaster*; Dr, zebrafish, *Danio rerio*; Gg, chicken, *Gallus gallus*; Mm, mouse, *Mus musculus*; Nv, sea anemone, *Nematostella vectensis*; Sp, sea urchin, *Strongylocentrotus purpuratus*. Click here for file

Additional file 3 **Sequence analysis of *AmSoxBb*.** (A) The nucleotide sequence and deduced amino acid sequence of the *AmSoxBb *cDNA. *AmSoxBb *cDNA contains a 1444 bp insert and an open reading frame (ORF) of 717 bp, corresponding to 239 amino acids. An asterisk indicates the stop codon. The 79 amino acids of HMG box sequence are highlighted in red. Numbers on the left side represent the nucleotide sequence; numbers on right side represent the amino acid sequence. A putative polyadenylation site is underlined. (B) Boxshade alignment of *AmSoxBb *and other subgroup B2 *Sox *genes. The HMG domain is underlined in red. The group B motif is underlined in blue. Asterisks indicate the key residues of group B. The species names are abbreviated as follows; Am, coral, *Acropora millepora*; Ce, nematode, *Caenorhabditis elegans*; Ci, ascidian, *Ciona intestinalis*; Dm, fruit-fly, *Drosophila melanogaster*; Dr, zebrafish, *Danio rerio*; Gg, chicken, *Gallus gallus*; Mm, mouse, *Mus musculus*; Nv, sea anemone, *Nematostella vectensis*; Sp, sea urchin, *Strongylocentrotus purpuratus*. Click here for file

Additional file 4 **Sequence analysis of *AmSoxC*.** (A) The nucleotide sequence and deduced amino acid sequence of the *AmSoxC *cDNA. *AmSoxC *cDNA contains a 1384 bp insert and an open reading frame (ORF) of 876 bp, corresponding to 292 amino acids. The 79 amino acids of HMG box sequence are highlighted in red. An asterisk indicates the stop codon. Numbers on the left side represent the nucleotide sequence; numbers on right side represent the amino acid sequence. No typical polyadenylation site (AATAAA) is found upstream of the poly(A)-tail. (B) Boxshade alignment of *AmSoxC *and other group C *Sox *genes. The HMG domain is underlined in red. An asterisk indicates the key residue of group C. The highly conserved regions (i and ii) are underlined. The species names are abbreviated as follows; Am, coral, *Acropora millepora*; Ci, ascidian, *Ciona intestinalis*; Dm, fruit-fly, *Drosophila melanogaster*; Dr, zebrafish, *Danio rerio*; Fr, Japanese pufferfish, *Fugu rubripes*; Gg, chicken, *Gallus gallus*; Hs, human, *Homo sapiens*; Nv, sea anemone, *Nematostella vectensis*. Click here for file

Additional file 5 **Sequence analysis of *AmSoxE1*.** (A) The nucleotide sequence and deduced amino acid sequence of the *AmSoxE1 *cDNA. *AmSoxE1 *cDNA contains a 1974 bp insert and an open reading frame (ORF) of 1224 bp, corresponding to 408 amino acids. An asterisk indicates the stop codon. The 79 amino acids of HMG box sequence are shaded in red. Numbers on the left side represent the nucleotide sequence; numbers on right side represent the amino acid sequence. Putative polyadenylation sites are underlined. (B) Boxshade alignment of *AmSoxE1 *and other group E *Sox *genes. The HMG domain is underlined in red. Asterisks indicate the key residues of group E. The highly conserved regions of group E (i, ii and iii) are underlined. The species names are abbreviated as follows; Am, coral, *Acropora millepora*; Ci, ascidian, *Ciona intestinalis*; Dm, fruit-fly, *Drosophila melanogaster*; Dr, zebrafish, *Danio rerio*; Fr, Japanese pufferfish, *Fugu rubripes*; Hs, human, *Homo sapiens*; Mm, mouse, *Mus musculus*; Nv, sea anemone, *Nematostella vectensis*. Click here for file

Additional file 6 **Sequence analysis of *AmSoxF*. **(A) The nucleotide sequence and deduced amino acid sequence of the *AmSoxF *cDNA. *AmSoxF *cDNA contains a 1637 bp insert and an open reading frame (ORF) of 1110 bp, corresponding to 370 amino acids. An asterisk indicates the stop codon. The 79 amino acids of HMG box sequence are shaded in red. Numbers on the left side represent the nucleotide sequence; numbers on right side represent the amino acid sequence. A putative polyadenylation site is underlined. (B) Boxshade alignment of *AmSoxF *and other group F *Sox *genes. The HMG domain is underlined in red. Asterisks indicate the key residues of group F. The highly conserved region of group F (transactivation domain) is underlined. The species names are abbreviated as follows; Am, coral, *Acropora millepora*; Ci, ascidian, *Ciona intestinalis*; Dm, fruit-fly, *Drosophila melanogaster*; Fr, Japanese pufferfish, *Fugu rubripes*; Hs, human, Homo sapiens; Mm, mouse, *Mus musculus*; Nv, sea anemone, *Nematostella vectensis*; Xl, frog, *Xenopus laevis*. Click here for file

Additional file 7 **Alignment of the HMG domains of SoxB proteins.** The asterisk indicates the position of the single amino acid residue insertion (position 74) in the aligned HMG domains of several cnidarian SoxB proteins. Species names are abbreviated as follows; Am, coral, *Acropora millepora*; Amq, sponge, *Amphimedon queenslandica*; Ce, nematode, *Caenorhabditis elegans*; Dm, fruit-fly, *Drosophila melanogaster*; Mm, mouse, *Mus musculus*; Nv, sea anemone, *Nematostella vectensis*. Click here for file

Additional file 8 **Phylogenetic analysis of the HMG domains in Sox proteins from non-Bilateria and the full human Sox repertoire.** The Maximum Likelihood tree shown was generated by MolPhy version 2.3 [49] using the Dayhoff model of protein evolution and local rearrangement of the NJ trees (1,000 bootstraps). *Acropora millepora Sox *genes are shown in red, whilst *Nematostella vectensis *genes are shown in blue. Species names are abbreviated as follows; Am, coral, *Acropora millepora*; Amq, demosponge, *Amphimedon queenslandica*; Bb, Japanese lancelet, *Branchiostoma belcheri*; Ce, nematode, *Caenorhabditis elegans*; Ci, ascidian, *Ciona intestinalis*; Dm, fruit-fly, *Drosophila melanogaster*; Hs, human, *Homo sapiens*; Hv, hydra, *Hydra vulgaris*; Mm, mouse, *Mus musculus*; Nc, red bread mold, *Neurospora crassa*; Nv, sea anemone, *Nematostella vectensis*; Om, rainbow trout, *Oncorhynchus mykiss*; Pm, sea lamprey, *Petromyzon marinus*; Sk, hemichordate, *Saccoglossus Kowalevskii*; Sp, sea urchin, *Strongylocentrotus purpuratus*; Tc, red flour beetle, *Tribolium castaneum*; ye-, yeast, *Schizosaccharomyces pombe*. Click here for file

Additional file 9 **Phylogenetic analysis of the HMG domains in *Acropora *and *Nematostella *Sox proteins.** The Maximum Likelihood tree shown was generated by MolPhy version 2.3 [49] using the Dayhoff model of protein evolution and local rearrangement of the NJ trees (1,000 bootstraps). All *Nematostella *Sox data reported in [9] were included in this analysis. Species names are abbreviated as follows; Am, coral, *Acropora millepora*; Amq, sponge, *Amphimedon queenslandica*; Bb, Japanese lancelet, *Branchiostoma belcheri*; Ce, nematode, *Caenorhabditis elegans*; Ci, ascidian, *Ciona intestinalis*; Dm, fruit-fly, *Drosophila melanogaster*; Dr, zebrafish, *Danio rerio*; Fr, Japanese pufferfish, *Fugu rubripes*; Gg, chicken, *Gallus gallus*; Hs, human, *Homo sapiens*; Hv, hydra, *Hydra vulgaris*; Mm, mouse, *Mus musculus*; Nc, red bread mold, *Neurospora crassa*; Nv, sea anemone, *Nematostella vectensis*; Om, rainbow trout, *Oncorhynchus mykiss*; Pm, sea lamprey, *Petromyzon marinus*; Sk, hemichordate, *Saccoglossus kowalevskii*; Sp, sea urchin, *Strongylocentrotus purpuratus*; Tc, red flour beetle, *Tribolium castaneum*; Xl, frog, *Xenopus laevis*; ye-, yeast, *Schizosaccharomyces pombe*. Click here for file
