# Supplementary material for: Sox genes in the coral Acropora millepora: divergent expression patterns reflect differences in developmental mechanisms within the Anthozoa
Source: BMC Evol Biol. 2008 Nov 12;8:311. doi: 10.1186/1471-2148-8-311 (PMC2613919; doi:10.1186/1471-2148-8-311)
Supplement: Additional file 1 — Sequence analysis of AmSoxB1. (A) The nucleotide sequence and deduced amino acid sequence of the AmSoxB1 cDNA. The 1888 bp AmSoxB1 cDNA contains an open reading frame (ORF) of 1008 bp, corresponding to 336 amino acids. An asterisk indicates the stop codon. The 79 amino acids of HMG box sequence are highlighted in red. Numbers on the left side represent the nucleotide sequence; numbers on right side represent the amino acid sequence. A putative polyadenylation site is underlined. (B) Boxshade alignment of AmSoxB1 and other subgroup B1 Sox genes. The HMG domain is underlined in red. The group B motif is underlined in blue. Asterisks indicate the key residues of group B. Highly conserved regions (i and ii) are underlined. The species names are abbreviated as follows; Am, coral, Acropora millepora; Ce, nematode, Caenorhabditis elegans; Ci, ascidian, Ciona intestinalis; Dm, fruit-fly, Drosophila melanogaster; Mm, mouse, Mus musculus; Nv, sea anemone, Nematostella vectensis; Sk, hemichordate, Saccoglossus kowalevskii; Sp, sea urchin, Strongylocentrotus purpuratus; Xl, frog, Xenopus laevis. [file 1471-2148-8-311-S1.pdf]

A

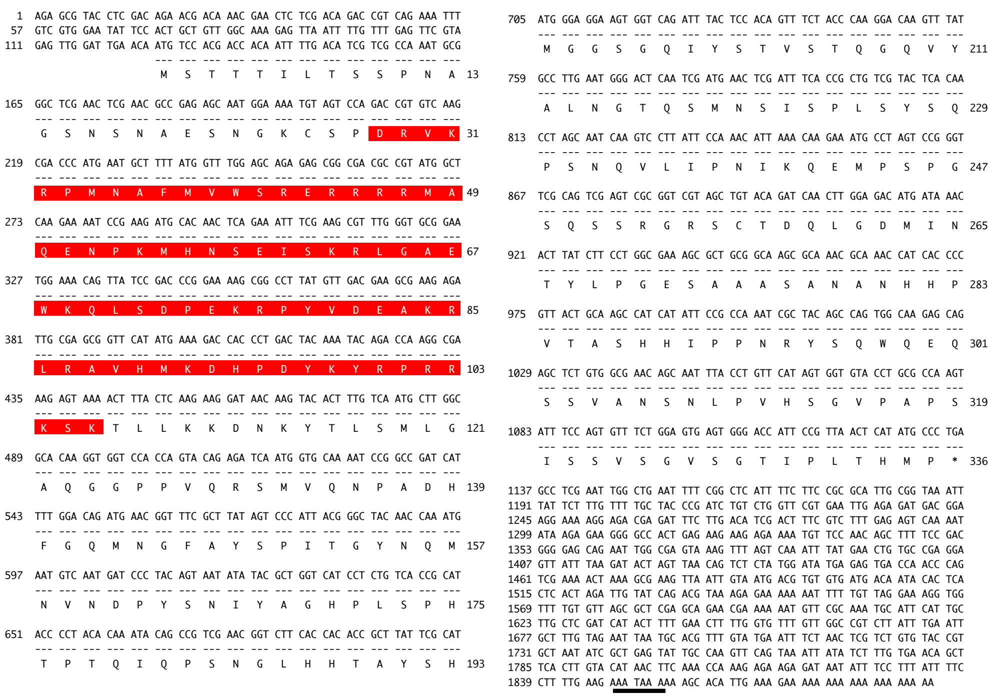

B

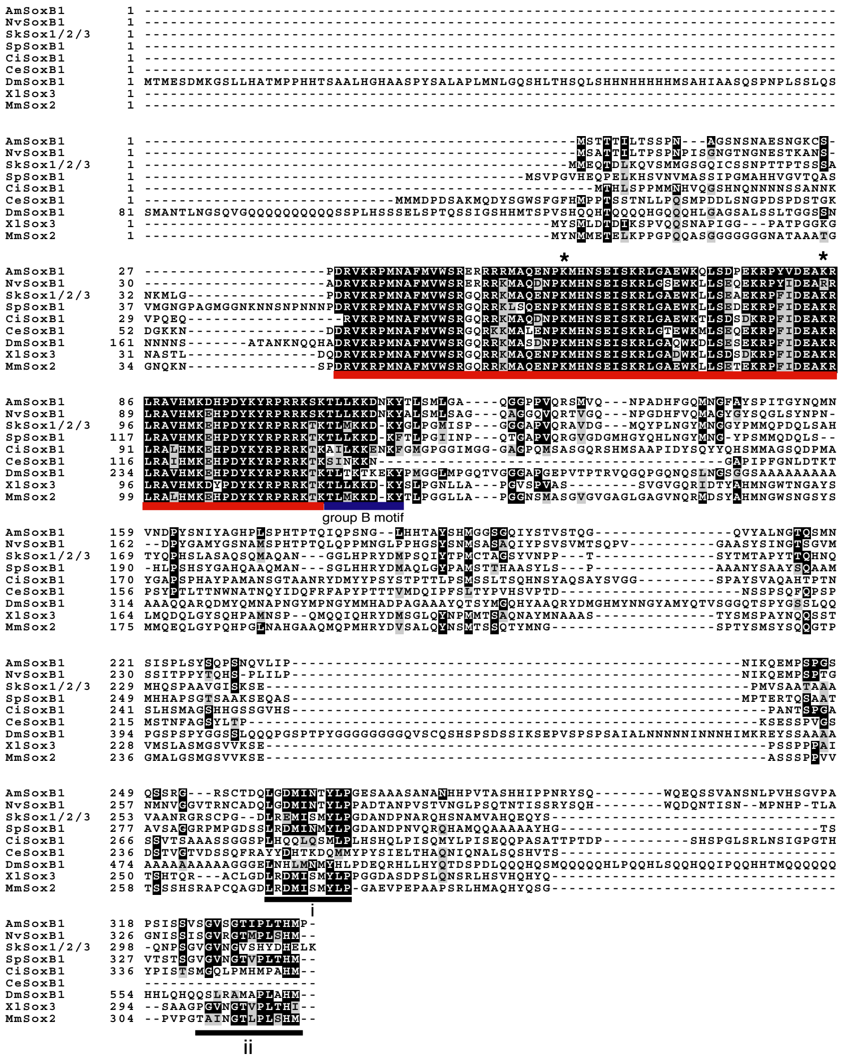
AmSoxB1
NvSoxB1
SkSox1/2/3
SpSoxB1
CiSoxB1
CeSoxB1
DmSoxB1
XlSox3
MmSox2
group B motif
i
ii
