# Supplementary material for: Sox genes in the coral Acropora millepora: divergent expression patterns reflect differences in developmental mechanisms within the Anthozoa
Source: BMC Evol Biol. 2008 Nov 12;8:311. doi: 10.1186/1471-2148-8-311 (PMC2613919; doi:10.1186/1471-2148-8-311)
Supplement: Additional file 2 — Sequence analysis of AmSoxBa. (A) The nucleotide sequence and deduced amino acid sequence of the AmSoxBa cDNA. The 79 amino acids of HMG box sequence are highlighted in red. Numbers on the left side represent the nucleotide sequence; numbers on right side represent the amino acid sequence. (B) Boxshade alignment of AmSoxBa and other subgroup B2 Sox genes. The HMG domain is underlined in red. The group B motif is underlined in blue. Asterisks indicate the key residues of group B. The species names are abbreviated as follows; Am, coral, Acropora millepora; Ce, nematode, Caenorhabditis elegans; Ci, ascidian, Ciona intestinalis; Dm, fruit-fly, Drosophila melanogaster; Dr, zebrafish, Danio rerio; Gg, chicken, Gallus gallus; Mm, mouse, Mus musculus; Nv, sea anemone, Nematostella vectensis; Sp, sea urchin, Strongylocentrotus purpuratus. [file 1471-2148-8-311-S2.pdf]

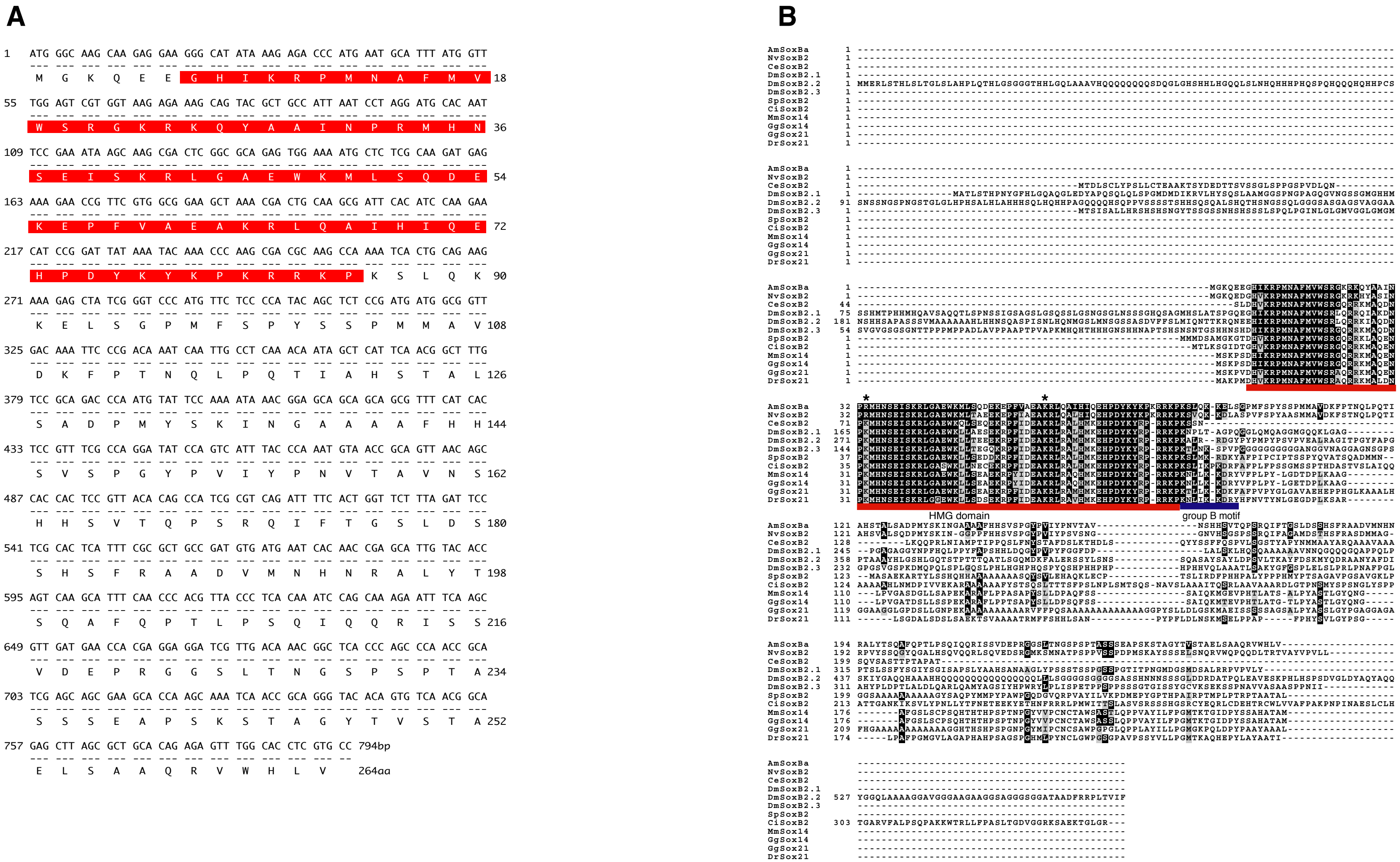
A
1 ATG GGC AAG CAA GAG GAA GGG CAT ATA AAG AGA CCC ATG AAT GCA TTT ATG GTT
M G K Q E E G H I K R P M N A F M V 18
55 TGG AGT CGT GGT AAG AGA AAG CAG TAC GCT GCC ATT AAT CCT AGG ATG CAC AAT
W S R G K R K Q Y A A I N P R M H N 36
109 TCC GAA ATA AGC AAG CGA CTC GGC GCA GAG TGG AAA ATG CTC TCG CAA GAT GAG
S E I S K R L G A E W K M L S Q D E 54
163 AAA GAA CCG TTC GTG GCG GAA GCT AAA CGA CTG CAA GCG ATT CAC ATC CAA GAA
K E P F V A E A K R L Q A I H I Q E 72
217 CAT CCG GAT TAT AAA TAC AAA CCC AAG CGA CGC AAG CCA AAA TCA CTG CAG AAG
H P D Y K Y K P K R R K P K S L Q K 90
271 AAA GAG CTA TCG GGT CCC ATG TTC TCC CCA TAC AGC TCT CCG ATG ATG GCG GTT
K E L S G P M F S P Y S S P M M A V 108
325 GAC AAA TTC CCG ACA AAT CAA TTG CCT CAA ACA ATA GCT CAT TCA ACG GCT TTG
D K F P T N Q L P Q T I A H S T A L 126
379 TCC GCA GAC CCA ATG TAT TCC AAA ATA AAC GGA GCA GCA GCA GCG TTT CAT CAC
S A D P M Y S K I N G A A A A F H H 144
433 TCC GTT TCG CCA GGA TAT CCA GTC ATT TAC CCA AAT GTA ACC GCA GTT AAC AGC
S V S P G Y P V I Y P N V T A V N S 162
487 CAC CAC TCC GTT ACA CAG CCA TCG CGT CAG ATT TTC ACT GGT TCT TTA GAT TCC
H H S V T Q P S R Q I F T G S L D S 180
541 TCG CAC TCA TTT CGC GCT GCC GAT GTG ATG AAT CAC AAC CGA GCA TTG TAC ACC
S H S F R A A D V M N H N R A L Y T 198
595 AGT CAA GCA TTT CAA CCC ACG TTA CCC TCA CAA ATC CAG CAA AGA ATT TCA AGC
S Q A F Q P T L P S Q I Q Q R I S S 216
649 GTT GAT GAA CCA CGA GGA GGA TCG TTG ACA AAC GGC TCA CCC AGC CCA ACC GCA
V D E P R G G S L T N G S P S P T A 234
703 TCG AGC AGC GAA GCA CCA AGC AAA TCA ACC GCA GGG TAC ACA GTG TCA ACG GCA
S S S E A P S K S T A G Y T V S T A 252
757 GAG CTT AGC GCT GCA CAG AGA GTT TGG CAC CTC GTG CC 794bp
E L S A A Q R V W H L V 264aa
B
AmSoxBa
NvSoxB2
CeSoxB2
DmSoxB2.1
DmSoxB2.2
DmSoxB2.3
SpSoxB2
CiSoxB2
MmSox14
GgSox14
GgSox21
DrSox21
HMG domain
group B motif
