# Supplementary material for: Sox genes in the coral Acropora millepora: divergent expression patterns reflect differences in developmental mechanisms within the Anthozoa
Source: BMC Evol Biol. 2008 Nov 12;8:311. doi: 10.1186/1471-2148-8-311 (PMC2613919; doi:10.1186/1471-2148-8-311)
Supplement: Additional file 3 — Sequence analysis of AmSoxBb. (A) The nucleotide sequence and deduced amino acid sequence of the AmSoxBb cDNA. AmSoxBb cDNA contains a 1444 bp insert and an open reading frame (ORF) of 717 bp, corresponding to 239 amino acids. An asterisk indicates the stop codon. The 79 amino acids of HMG box sequence are highlighted in red. Numbers on the left side represent the nucleotide sequence; numbers on right side represent the amino acid sequence. A putative polyadenylation site is underlined. (B) Boxshade alignment of AmSoxBb and other subgroup B2 Sox genes. The HMG domain is underlined in red. The group B motif is underlined in blue. Asterisks indicate the key residues of group B. The species names are abbreviated as follows; Am, coral, Acropora millepora; Ce, nematode, Caenorhabditis elegans; Ci, ascidian, Ciona intestinalis; Dm, fruit-fly, Drosophila melanogaster; Dr, zebrafish, Danio rerio; Gg, chicken, Gallus gallus; Mm, mouse, Mus musculus; Nv, sea anemone, Nematostella vectensis; Sp, sea urchin, Strongylocentrotus purpuratus. [file 1471-2148-8-311-S3.pdf]

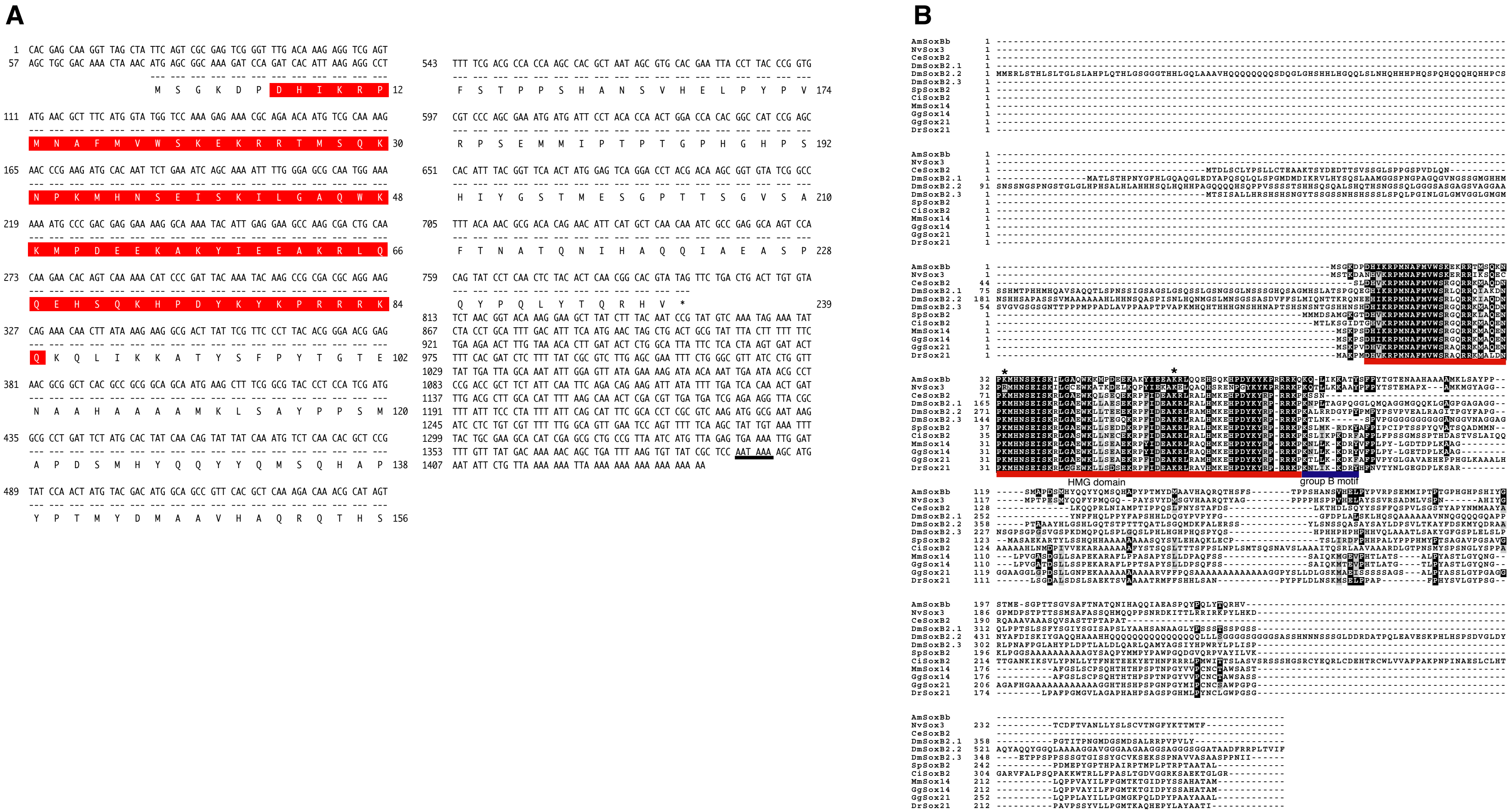
A
B
HMG domain
group B motif
