# Supplementary material for: Sox genes in the coral Acropora millepora: divergent expression patterns reflect differences in developmental mechanisms within the Anthozoa
Source: BMC Evol Biol. 2008 Nov 12;8:311. doi: 10.1186/1471-2148-8-311 (PMC2613919; doi:10.1186/1471-2148-8-311)
Supplement: Additional file 4 — Sequence analysis of AmSoxC. (A) The nucleotide sequence and deduced amino acid sequence of the AmSoxC cDNA. AmSoxC cDNA contains a 1384 bp insert and an open reading frame (ORF) of 876 bp, corresponding to 292 amino acids. The 79 amino acids of HMG box sequence are highlighted in red. An asterisk indicates the stop codon. Numbers on the left side represent the nucleotide sequence; numbers on right side represent the amino acid sequence. No typical polyadenylation site (AATAAA) is found upstream of the poly(A)-tail. (B) Boxshade alignment of AmSoxC and other group C Sox genes. The HMG domain is underlined in red. An asterisk indicates the key residue of group C. The highly conserved regions (i and ii) are underlined. The species names are abbreviated as follows; Am, coral, Acropora millepora; Ci, ascidian, Ciona intestinalis; Dm, fruit-fly, Drosophila melanogaster; Dr, zebrafish, Danio rerio; Fr, Japanese pufferfish, Fugu rubripes; Gg, chicken, Gallus gallus; Hs, human, Homo sapiens; Nv, sea anemone, Nematostella vectensis. [file 1471-2148-8-311-S4.pdf]

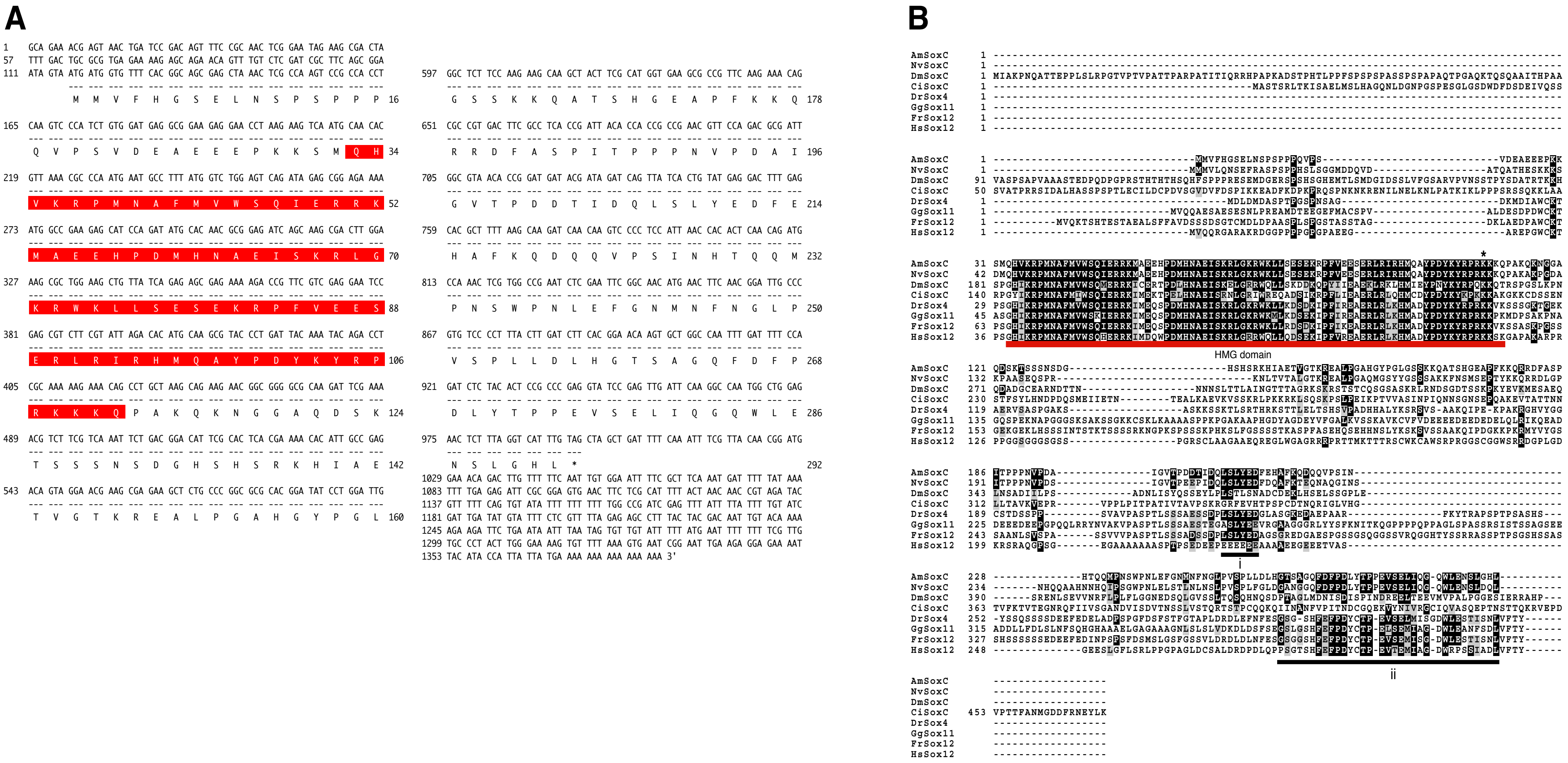
A
B
HMG domain
i
ii
