# Supplementary material for: Sox genes in the coral Acropora millepora: divergent expression patterns reflect differences in developmental mechanisms within the Anthozoa
Source: BMC Evol Biol. 2008 Nov 12;8:311. doi: 10.1186/1471-2148-8-311 (PMC2613919; doi:10.1186/1471-2148-8-311)
Supplement: Additional file 5 — Sequence analysis of AmSoxE1. (A) The nucleotide sequence and deduced amino acid sequence of the AmSoxE1 cDNA. AmSoxE1 cDNA contains a 1974 bp insert and an open reading frame (ORF) of 1224 bp, corresponding to 408 amino acids. An asterisk indicates the stop codon. The 79 amino acids of HMG box sequence are shaded in red. Numbers on the left side represent the nucleotide sequence; numbers on right side represent the amino acid sequence. Putative polyadenylation sites are underlined. (B) Boxshade alignment of AmSoxE1 and other group E Sox genes. The HMG domain is underlined in red. Asterisks indicate the key residues of group E. The highly conserved regions of group E (i, ii and iii) are underlined. The species names are abbreviated as follows; Am, coral, Acropora millepora; Ci, ascidian, Ciona intestinalis; Dm, fruit-fly, Drosophila melanogaster; Dr, zebrafish, Danio rerio; Fr, Japanese pufferfish, Fugu rubripes; Hs, human, Homo sapiens; Mm, mouse, Mus musculus; Nv, sea anemone, Nematostella vectensis. [file 1471-2148-8-311-S5.pdf]

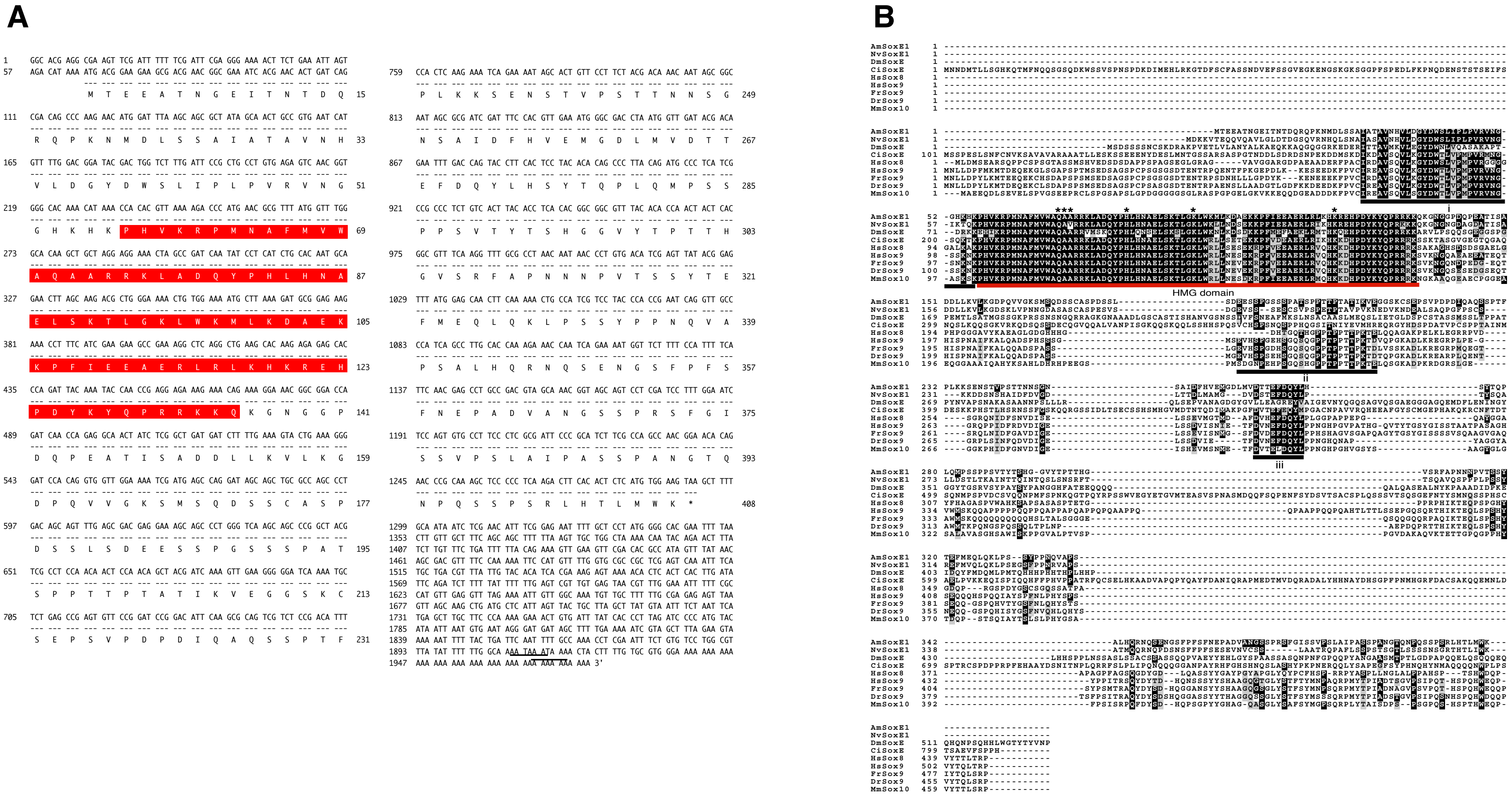
A
B
HMG domain
i
ii
iii
