# Supplementary material for: Sox genes in the coral Acropora millepora: divergent expression patterns reflect differences in developmental mechanisms within the Anthozoa
Source: BMC Evol Biol. 2008 Nov 12;8:311. doi: 10.1186/1471-2148-8-311 (PMC2613919; doi:10.1186/1471-2148-8-311)
Supplement: Additional file 6 — Sequence analysis of AmSoxF. (A) The nucleotide sequence and deduced amino acid sequence of the AmSoxF cDNA. AmSoxF cDNA contains a 1637 bp insert and an open reading frame (ORF) of 1110 bp, corresponding to 370 amino acids. An asterisk indicates the stop codon. The 79 amino acids of HMG box sequence are shaded in red. Numbers on the left side represent the nucleotide sequence; numbers on right side represent the amino acid sequence. A putative polyadenylation site is underlined. (B) Boxshade alignment of AmSoxF and other group F Sox genes. The HMG domain is underlined in red. Asterisks indicate the key residues of group F. The highly conserved region of group F (transactivation domain) is underlined. The species names are abbreviated as follows; Am, coral, Acropora millepora; Ci, ascidian, Ciona intestinalis; Dm, fruit-fly, Drosophila melanogaster; Fr, Japanese pufferfish, Fugu rubripes; Hs, human, Homo sapiens; Mm, mouse, Mus musculus; Nv, sea anemone, Nematostella vectensis; Xl, frog, Xenopus laevis. [file 1471-2148-8-311-S6.pdf]

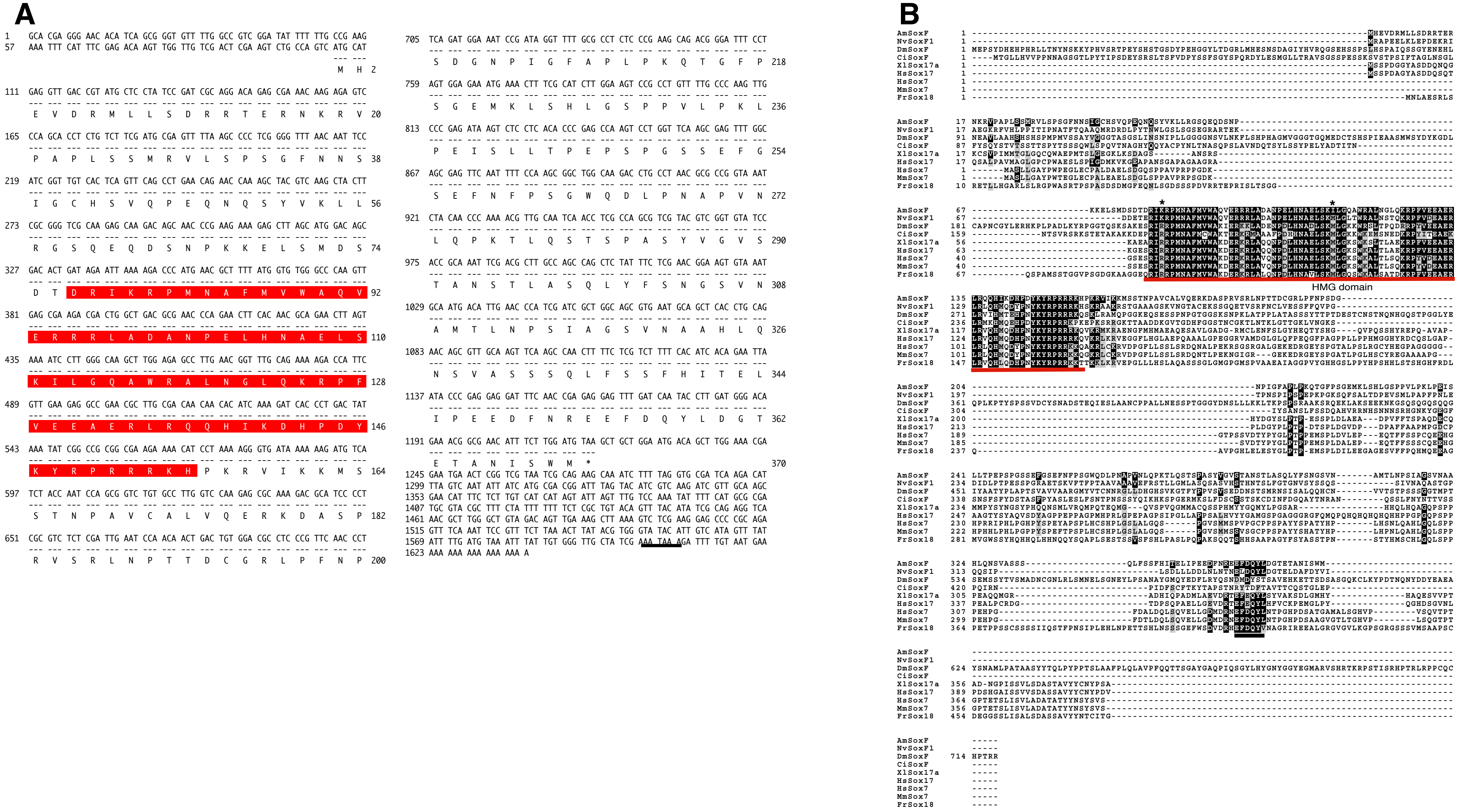
A
B
HMG domain
