# Supplementary material for: Sox genes in the coral Acropora millepora: divergent expression patterns reflect differences in developmental mechanisms within the Anthozoa
Source: BMC Evol Biol. 2008 Nov 12;8:311. doi: 10.1186/1471-2148-8-311 (PMC2613919; doi:10.1186/1471-2148-8-311)
Supplement: Additional file 7 — Alignment of the HMG domains of SoxB proteins. The asterisk indicates the position of the single amino acid residue insertion (position 74) in the aligned HMG domains of several cnidarian SoxB proteins. Species names are abbreviated as follows; Am, coral, Acropora millepora; Amq, sponge, Amphimedon queenslandica; Ce, nematode, Caenorhabditis elegans; Dm, fruit-fly, Drosophila melanogaster; Mm, mouse, Mus musculus; Nv, sea anemone, Nematostella vectensis. [file 1471-2148-8-311-S7.pdf]

```
                                                                            *
AmSoxB1      DRVKRPMNAFMVWSRERRRRMAQENPKMHNSEISKRLGAEWKQLSDPEKRPYVDEAKRLRAVHMKDHPDYKYRP-RRKSK
NvSoxB1      DRVKRPMNAFMVWSRERRRKMAQDNPKMHNSEISKRLGSEWKLLSEQEKRPYIDEARRLRAVHMKEHPDYKYRP-RRKSK
NvSox1       EHVKRPMNAFMVWSREERRKIAQENPKMHNSEISKRLGSEWKQLADDDKKPFVEEAKKLRAQHMKEHPDYKYRP-RRMPK
AmSoxBa      GHIKRPMNAFMVWSRGKRKQYAAINPRMHNSEISKRLGAEWKMLSQDEKEPFVAEAKRLQAIHIQEHPDYKYKPKRRKPK
NvSoxB2      GHVKRPMNAFMVWSRGKRKHYASINPRMHNSEISKRLGAEWKMLTAEEKEPFIAEAKRLQALHIQEHPDYKYKPKRRKPK
NvSox2       DHIKRPMNAYMVWSRKERRRIAEECPRMLNSEISKRLGLEWNSLTLDEKQPYVEEAKRLRELHKKDHPDYKYQP-KRKPK
AmSoxBb      DHIKRPMNAFMVWSKEKRRTMSQKNPKMHNSEISKILGAQWKKMPDEEKAKYIEEAKRLQQEHSQKHPDYKYKPRRRKQK
NvSox3       NHVKRPMNAFMVWSKERRRIKSQECPRMHNSEISKILGCEWKATKDELKQPYIEKAKELQAQHSRENPGYKYKPRRRKPK
AmqSoxB1     DKVKRPMNAFMVWSRKMRKKIADENPKMHNSEISKRLGTQWKALSEEDKRPFIDEAKRLREAHMKKHPNYKYKP-KRKKQ
AmqSoxB2     DHIKRPMNAFMVWSKERRKELAQENPRMHNSELSKKLGAEWKALSDTNKHRYIEEAKKIREQHMAEFPHYRYRP-RRKPK
DmSoxB1      DRVKRPMNAFMVWSRGQRRKMASDNPKMHNSEISKRLGAQWKDLSESEKRPFIDEAKRLRAVHMKEHPDYKYRP-RRKTK
CeSoxB1      DRVKRPMNAFMVWSRGQRKKMALENPKMHNSEISKRLGTEWKMLSEQEKRPFIDEAKRLRAIHMKEHPDYKYRP-RRKTK
MmSox2       DRVKRPMNAFMVWSRGQRRKMAQENPKMHNSEISKRLGAEWKLLSETEKRPFIDEAKRLRALHMKEHPDYKYRP-RRKTK
DmSoxB2.1    GHIKRPMNAFMVWSRLQRRQIAKDNPKMHNSEISKRLGAEWKLLAESEKRPFIDEAKRLRALHMKEHPDYKYRP-RRKPK
CeSoxB2      DHVKRPMNAFMVWSRGQRRKMAQDNPKMHNSEISKRLGAEWKQLSEQEKRPFIDEAKRLRALHMKEHPDYKYRP-RRKPK
MmSox14      DHIKRPMNAFMVWSRGQRRKMAQENPKMHNSEISKRLGAEWKLLSEAEKRPYIDEAKRLRAQHMKEHPDYKYRP-RRKPK
```
