# Supplementary figures and images for: Sox genes in the coral Acropora millepora: divergent expression patterns reflect differences in developmental mechanisms within the Anthozoa
Source: BMC Evol Biol. 2008 Nov 12;8:311. doi: 10.1186/1471-2148-8-311 (PMC2613919; doi:10.1186/1471-2148-8-311)

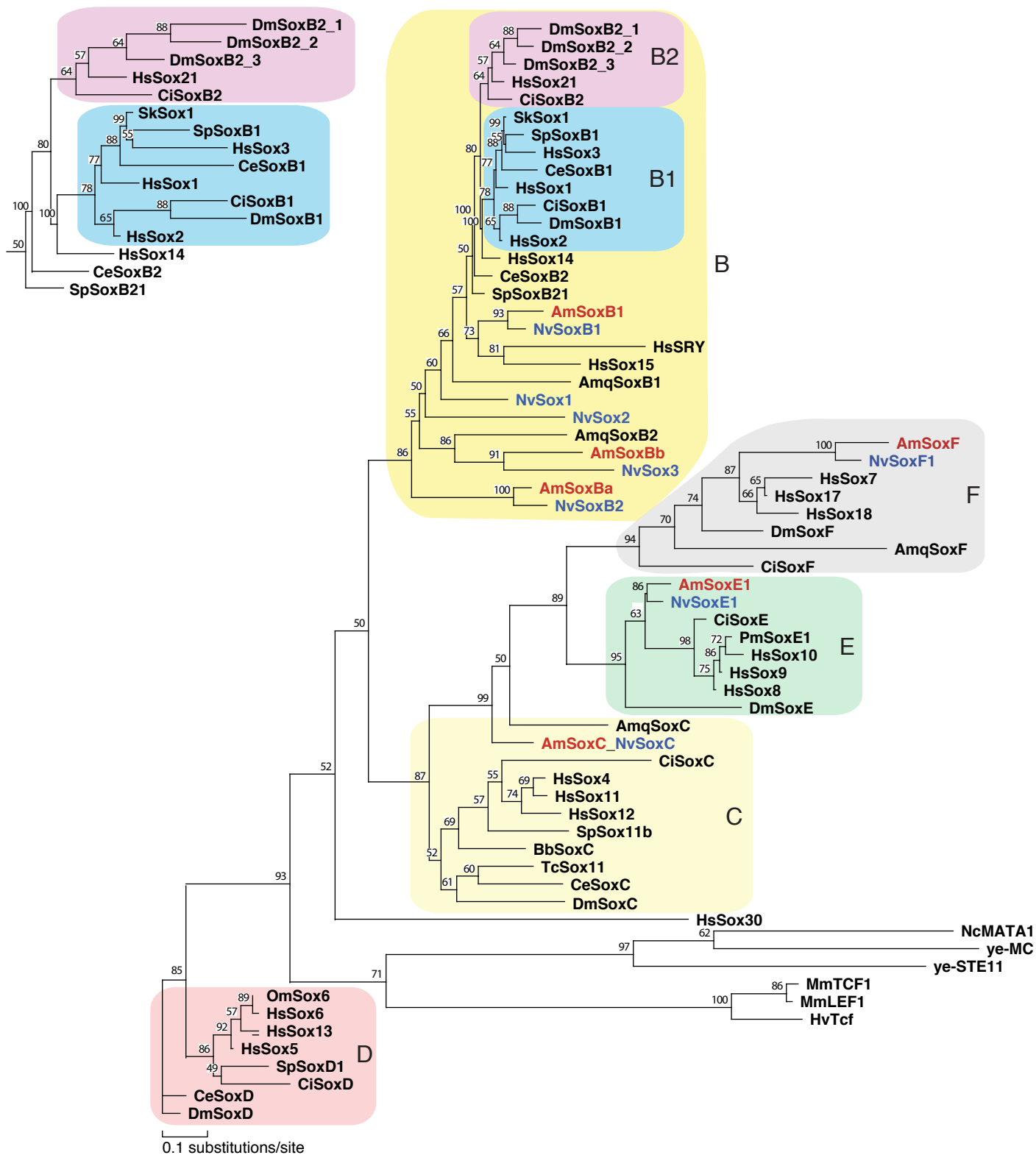

Supplement: Additional file 8 — Phylogenetic analysis of the HMG domains in Sox proteins from non-Bilateria and the full human Sox repertoire. The Maximum Likelihood tree shown was generated by MolPhy version 2.3 [49] using the Dayhoff model of protein evolution and local rearrangement of the NJ trees (1,000 bootstraps). Acropora millepora Sox genes are shown in red, whilst Nematostella vectensis genes are shown in blue. Species names are abbreviated as follows; Am, coral, Acropora millepora; Amq, demosponge, Amphimedon queenslandica; Bb, Japanese lancelet, Branchiostoma belcheri; Ce, nematode, Caenorhabditis elegans; Ci, ascidian, Ciona intestinalis; Dm, fruit-fly, Drosophila melanogaster; Hs, human, Homo sapiens; Hv, hydra, Hydra vulgaris; Mm, mouse, Mus musculus; Nc, red bread mold, Neurospora crassa; Nv, sea anemone, Nematostella vectensis; Om, rainbow trout, Oncorhynchus mykiss; Pm, sea lamprey, Petromyzon marinus; Sk, hemichordate, Saccoglossus Kowalevskii; Sp, sea urchin, Strongylocentrotus purpuratus; Tc, red flour beetle, Tribolium castaneum; ye-, yeast, Schizosaccharomyces pombe. [file 1471-2148-8-311-S8.pdf]

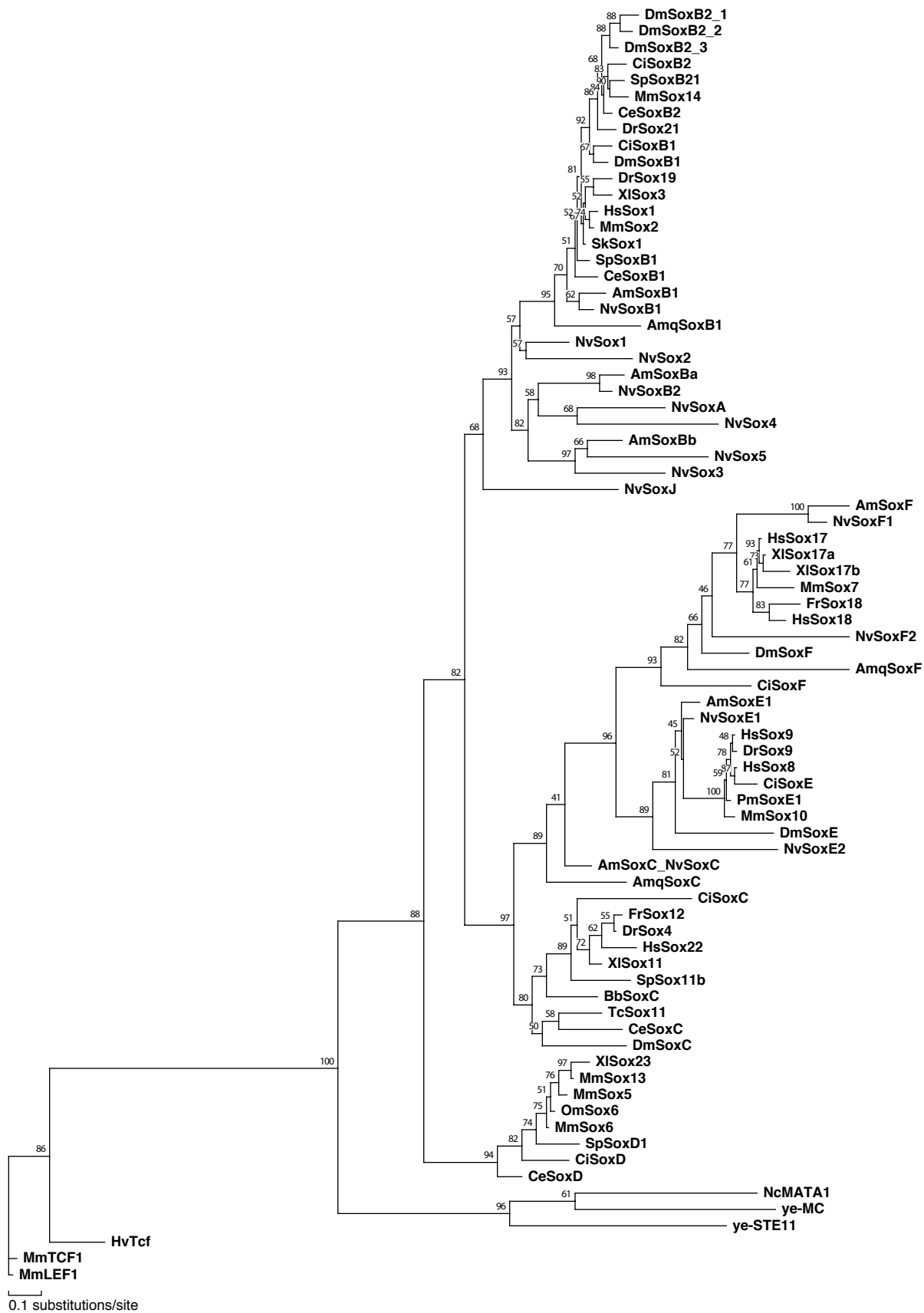

Supplement: Additional file 9 — Phylogenetic analysis of the HMG domains in Acropora and Nematostella Sox proteins. The Maximum Likelihood tree shown was generated by MolPhy version 2.3 [49] using the Dayhoff model of protein evolution and local rearrangement of the NJ trees (1,000 bootstraps). All Nematostella Sox data reported in [9] were included in this analysis. Species names are abbreviated as follows; Am, coral, Acropora millepora; Amq, sponge, Amphimedon queenslandica; Bb, Japanese lancelet, Branchiostoma belcheri; Ce, nematode, Caenorhabditis elegans; Ci, ascidian, Ciona intestinalis; Dm, fruit-fly, Drosophila melanogaster; Dr, zebrafish, Danio rerio; Fr, Japanese pufferfish, Fugu rubripes; Gg, chicken, Gallus gallus; Hs, human, Homo sapiens; Hv, hydra, Hydra vulgaris; Mm, mouse, Mus musculus; Nc, red bread mold, Neurospora crassa; Nv, sea anemone, Nematostella vectensis; Om, rainbow trout, Oncorhynchus mykiss; Pm, sea lamprey, Petromyzon marinus; Sk, hemichordate, Saccoglossus kowalevskii; Sp, sea urchin, Strongylocentrotus purpuratus; Tc, red flour beetle, Tribolium castaneum; Xl, frog, Xenopus laevis; ye-, yeast, Schizosaccharomyces pombe. [file 1471-2148-8-311-S9.pdf]
